# Stability in the South, Turbulence Toward the North: Evolutionary History of *Aurinia saxatilis* (Brassicaceae) Revealed by Phylogenomic and Climatic Modelling Data

**DOI:** 10.3389/fpls.2022.822331

**Published:** 2022-03-14

**Authors:** Ivana Rešetnik, Eliška Záveská, Marin Grgurev, Sandro Bogdanović, Paolo Bartolić, Božo Frajman

**Affiliations:** ^1^Department of Biology, Faculty of Science, University of Zagreb, Zagreb, Croatia; ^2^Institute of Botany, Czech Academy of Sciences, Prague, Czechia; ^3^Department of Agricultural Botany, Faculty of Agriculture, University of Zagreb, Zagreb, Croatia; ^4^Centre of Excellence for Biodiversity and Molecular Plant Breeding, Zagreb, Croatia; ^5^Department of Botany, Charles University, Prague, Czechia; ^6^Department of Botany, University of Innsbruck, Innsbruck, Austria

**Keywords:** *Aurinia saxatilis*, demographic modelling, glacial refugia, *ndh*F, RAD sequencing, species distribution modelling

## Abstract

The Balkan Peninsula played an important role in the evolution of many Mediterranean plants and served as a major source for post-Pleistocene colonisation of central and northern Europe. Its complex geo-climatic history and environmental heterogeneity significantly influenced spatiotemporal diversification and resulted in intricate phylogeographic patterns. To explore the evolutionary dynamics and phylogeographic patterns within the widespread eastern Mediterranean and central European species *Aurinia saxatilis*, we used a combination of phylogenomic (restriction-site associated DNA sequencing, RADseq) and phylogenetic (sequences of the plastid marker *ndh*F) data as well as species distribution models generated for the present and the Last Glacial Maximum (LGM). The inferred phylogenies retrieved three main geographically distinct lineages. The southern lineage is restricted to the eastern Mediterranean, where it is distributed throughout the Aegean area, the southern Balkan Peninsula, and the southern Apennine Peninsula, and corresponds to the species main distribution area during the LGM. The eastern lineage extends from the eastern Balkan Peninsula over the Carpathians to central Europe, while the central lineage occupies the central Balkan Peninsula. Molecular dating places the divergence among all the three lineages to the early to middle Pleistocene, indicating their long-term independent evolutionary trajectories. Our data revealed an early divergence and stable *in situ* persistence of the southernmost, eastern Mediterranean lineage, whereas the mainland, south-east European lineages experienced more complex and turbulent evolutionary dynamics triggered by Pleistocene climatic oscillations. Our data also support the existence of multiple glacial refugia in southeast Europe and highlight the central Balkan Peninsula not only as a cradle of lineage diversifications but also as a source of lineage dispersal. Finally, the extant genetic variation within *A. saxatilis* is congruent with the taxonomic separation of peripatric *A. saxatilis* subsp. *saxatilis* and *A. saxatilis* subsp. *orientalis*, whereas the taxonomic status of *A. saxatilis* subsp. *megalocarpa* remains doubtful.

## Introduction

The Balkan, Apennine and Iberian Peninsulas extending to the Mediterranean Basin represent European biodiversity hotspots, hosting high species richness and genetic diversity (Petit et al., 2003; Hewitt, 2011; Nieto Feliner, 2014; Gömöry et al., 2020). They were important Pleistocene refugia, from where biota expanded during interglacial periods, including the Holocene, and colonised central and northern Europe (Taberlet et al., 1998; Hewitt, 1999; Tzedakis et al., 2013). Especially, the Balkan Peninsula as part of the eastern Mediterranean is standing out in its role as a source for postglacial colonisation of Europe (Hewitt, 2011; Nieto Feliner, 2014). A lower impact of the Ice Ages coupled with climatic and topographic complexity of the Balkans facilitated spatiotemporal processes, enabling glacial survival and triggered a differentiation of biota. Some plants experienced stable long-term persistence without extensive differentiation and range expansion during the Holocene (e.g., *Aesculus hippocastanum*, Walas et al., 2019; *Euphorbia heldreichii*, Caković and Frajman, 2020), while other groups underwent enhanced population differentiation and speciation. Recent phylogeographic studies focusing on the southern Balkan Peninsula and adjacent Aegean Basin revealed the importance of this area for genetic diversification and speciation and indicated its marginal role in northward expansion, as seen in the *Alyssum montanum-repens* complex (Španiel et al., 2017), annual *Alyssum* species (Cetlová et al., 2021), *Campanula* (Crowl et al., 2015), *Cymbalaria* (Carnicero et al., 2020), the *Euphorbia verrucosa* alliance (Caković et al., 2021) and the genus *Nigella* (Jaros et al., 2018). Similarly, in *Veronica chamaedrys* (Bardy et al., 2010) and *Edraianthus graminifolius* (Surina et al., 2014), southern populations exhibited deeper and older differentiation not followed by lineage expansions. On the other hand, several more northern lineages successfully expanded from the Balkan Peninsula and colonised central Europe and areas beyond (Magri et al., 2006; Frajman and Oxelman, 2007; Bardy et al., 2010; Rešetnik et al., 2016; Đurović et al., 2017; Caković et al., 2021). In addition, multiple Balkan lineages migrated *trans*-Adriatically and colonised the Apennine Peninsula (e.g., Rešetnik et al., 2016; Frajman and Schönswetter, 2017; Falch et al., 2019) or expanded to the Carpathians and the Pontic area (e.g., Frajman and Oxelman, 2007; Puşcaş et al., 2008; Csergö et al., 2009; Ronikier, 2011; Stachurska-Swakoń et al., 2013; Đurović et al., 2017).

The initial simplistic scenario of exclusively southern peninsular refugia has been challenged because of growing evidence suggesting more complex patterns, including glacial survival in more northern European regions. ‘Cryptic’ northern refugia were not only inferred for cold-tolerant arctic, boreal, and subalpine species (e.g., Stachurska-Swakoń et al., 2013; Ronikier and Zalewska-Gałosz, 2014) but also for temperate species, such as *Arabidopsis arenosa* (Kolář et al., 2016), *Arabidopsis halleri* (Šrámková-Fuxová et al., 2017), *Cyclamen purpurascens* (Slovák et al., 2012), *Erythronium dens-canis* (Bartha et al., 2015), *Euphorbia ilirica* (Frajman et al., 2016), *Helleborus niger* (Záveská et al., 2021), *Hepatica transsilvanica* (Laczkó and Sramkó, 2020), and *Rosa pendulina* (Daneck et al., 2016). These studies imply that phylogeographic connections between the Balkan Peninsula and central Europe (Bardy et al., 2010; Slovák et al., 2012; Rešetnik et al., 2016; Záveská et al., 2021) are older than previously thought and not necessarily examples of postglacial range expansions but rather cases of local LGM survival in central European refugia.

*Aurinia saxatilis* (L.) Desv. is a species ranging from central Europe (northern Pannonia, Moravia) over the Carpathians to the central, eastern, and southern Balkan Peninsula and adjacent shores of western Asia Minor as well as the southern Apennine Peninsula (Persson, 1971). It is the most widely distributed of seven *Aurinia* species, which have, as yet, unresolved phylogenetic relationships (Rešetnik et al., 2013). It is an up to 30-cm tall evergreen perennial plant having rosette- and stem leaves with stellate indumentum and long branched inflorescence-bearing numerous small, bright yellow flowers. Seeds of this species are thin, rounded, and broadly winged, which can facilitate wind dispersal. It is a popular ornamental plant in gardens, with the common name Basket of Gold referring to its numerous bright yellow flowers. *Aurinia saxatilis* is a diploid species (Lysak et al., 2009), growing on calcareous rocky grounds and dry soils mainly as saxicolous chasmophyte-forming vegetation of walls, cliffs, and rocky places (Persson, 1971). Because of its morphological variability, it has been divided into three subspecies having partly overlapping distributions (Dudley, 1964; Persson, 1971; Akeroyd, 1993; Plazibat, 2009). Typical *A. saxatilis* is widespread in central and southeastern Europe, *A. saxatilis* subsp. *orientalis* (Ard.) T.R. Dudley is distributed in the Balkan Peninsula, southern Carpathians, and western Anatolia, whereas *A. saxatilis* subsp. *megalocarpa* (Hausskn.) T.R. Dudley is limited to the southern Apennine Peninsula and the Aegean region (including Crete and the west Anatolian coast).

To explore the evolutionary dynamics and phylogeographic patterns in *A. saxatilis*, here, we combine phylogenomics and species distribution modelling. First, using plastid *ndhF* sequences, we reconstruct phylogenetic relationships and establish a temporal outline of its diversification. Second, using genome-wide SNPs generated by restriction-site associated DNA sequencing (RADseq) coupled with demographic and species distribution modelling, we reveal range wide patterns of genetic diversity, compare alternative scenarios of population divergences, and infer the species’ evolutionary history. We further explore whether the distribution of *A. saxatilis* in eastern and central Europe is a consequence of post-glacial expansion from the southern Balkan refugium or if there is evidence for *in situ* glacial survival. Finally, we intersect the phylogenetic data with infraspecific entities and discuss taxonomic implications.

## Materials and Methods

### Plant Material

We sampled 77 populations of *A. saxatilis* throughout its range (Supplementary Figure 1). In addition to herbarium specimens, a leaf material of one to five individuals per population was collected in silica gel. Voucher data and GenBank numbers are presented in Supplementary Table 1, and the geographic origin of the sampled populations is shown in Supplementary Figure 1. For one individual from 75 populations, we sequenced the plastid *ndh*F region, and for one to three individuals from 63 populations, RADseq was performed. Additionally, in plastid analyses we used six published GenBank sequences of *Aurinia* and 56 of other Alysseae (Supplementary Table 2). For RADseq, we used the published genome of *Odontarrhena argentea* (BioSample: SAMEA4639485) as a reference for mapping raw reads and as an outgroup.

### Wet Lab Methods

Extraction of total genomic DNA was performed from silica gel-dried leaves using GenElute Plant Genomic DNA Miniprep Kit (Sigma–Aldrich) and following the manufacturer’s instructions. PCR and sequencing of *ndhF* were performed as described in Rešetnik et al. (2013) using the primers 5F, 989R, 989F, 1703R, 1410F, and 2100R (Beilstein et al., 2006). After checking amplicons on 1% TBE-agarose gel, they were purified enzymatically using exonuclease I and shrimp alkaline phosphatase (SAP; Fermentas) following the manufacturer’s instructions. Sequencing was carried out at Macrogen Europe using the same primers as for amplification. Single-digest RADseq libraries were prepared using the restriction enzyme *Pst*I (New England Biolabs) and a protocol adapted from Paun et al. (2016) and Záveská et al. (2021). Up to three individuals of each of 63 populations of *A. saxatilis* were sequenced on Illumina HiSeq at VBCF NGS Unit^1^ as 100-bp single-end reads.

### Plastid Data Analyses

Contigs of plastid sequences were assembled and edited, and the sequences were aligned using Geneious Pro 10.2.3^2^. Maximum parsimony (MP) and MP bootstrap (MPB) analyses were performed using PAUP 4.0b10 (Swofford, 2002). The most parsimonious trees were searched for heuristically with 1,000 replicates of random sequence addition, TBR swapping and MulTrees on. Swapping was performed on a maximum of 1,000 trees (nchuck = 1000). All characters were equally weighted and unordered. The dataset was bootstrapped using full heuristics, 1,000 replicates, TBR branch swapping, MulTrees option off, and random addition sequence with five replicates. *Berteroa incana*, *Berteroa obliqua*, *Galitzkya macrocarpa*, and *Lepidotrichum uechtritzianum* were used for rooting based on Rešetnik et al. (2013). Bayesian analyses were performed with the MrBayes 3.2.2 (Ronquist et al., 2012) applying the GTR + G substitution model proposed by the Akaike information criterion implemented in the MrModelTest (Nylander, 2004). The settings for the Metropolis-coupled Markov chain Monte Carlo process included two runs with four chains each, run simultaneously for 10,000,000 generations each, sampling trees every 1,000^th^ generation using default priors. The posterior probability (PP) of the phylogeny and its branches was determined from the combined set of trees, discarding the first 2,500 trees of each run as burn-in. Convergence of the MCMC procedure was assessed further by calculating effective sample sizes (ESSs) with the programme Tracer 1.6.0 (Rambaut et al., 2014). We also analysed the plastid sequences using statistical parsimony as implemented in TCS (Clement et al., 2000) with the connection limit set to 95% and gaps (all being 1 bp long) treated as fifth character state, because all the chromatograms were unambiguous, thus enabling the usage of gap characters in the TCS analysis.

Divergence times were estimated using the plastid sequences and following dating approaches of Huang et al. (2020) and Walden et al. (2020). More precisely, we selected four accessions representing major lineages of *A. saxatilis* as revealed by analyses of the complete plastid dataset (see above) and aligned them to sequences representing other *Aurinia* species and different outgroup genera as used in Huang et al. (2020) and Walden et al. (2020), respectively. In preliminary analyses we analysed both datasets, applying different secondary calibration points from both studies. In the first case, we set the age of the clade corresponding to tribe Alysseae to 13.8–19.9 Mya (95% values of highest posterior densities, HPDs) according to Huang et al. (2020), whereas in the second case, we set the age of this clade to 12.78–21.03 Mya following Walden et al. (2020). As both analyses resulted in comparable age estimations for *Aurinia*, here, we more precisely present only the analysis following Huang et al. (2020). Dating analysis was performed using the BEAST 1.8.2 (Drummond et al., 2012), applying the birth-death speciation prior (Gernhard, 2008) and the GTR + G substitution model with estimated base frequencies, and a lognormal relaxed clock with a weakly informative prior on clock rate (exponential with mean 0.001). The prior age of the root was set to 17.1 million years with a normally distributed standard deviation of 1.45, which corresponds to the median age and roughly to 95% HPD interval 13.8–19.9 Mya of the corresponding node inferred in Huang et al. (2020). Two MCMC chains were run for 100 million generations, logging parameters every 10,000 generations. The performance of the analysis was checked with Tracer 1.6.0 (Rambaut et al., 2014); both the effective sample sizes and mixing were deemed appropriate. Maximum clade credibility (MCC) trees were produced and annotated with Tree Annotator (part of the BEAST package) after removing 10% burn-in and combining the two tree files with the LogCombiner (part of the BEAST package), and visualised with the FigTree 1.4.2 (Rambaut, 2014).

### RADseq: Variant Calling and Filtering

The raw reads were demultiplexed according to index barcodes using the BamIndexDecoder 1.03^3^ and based on inline barcodes with process_radtags.pl implemented in the Stacks 1.35 (Catchen et al., 2011, 2013) with default settings. We used an Illumina short-read assembly of *O. argentea* (BioSample: SAMEA4639485) as a reference for mapping the raw reads of *A. saxatilis* samples with the mem algorithm of the BWA 0.7.5a-r405 (Li and Durbin, 2009). Mapping files were then sorted by reference coordinates, and read groups were added with the Picard Tools 2.0.1^4^. Realignments around indels were performed for each bam file using the Genome Analysis Toolkit 3.6-0-g89b7209 (McKenna et al., 2010). We conducted the realignments around indels to correct mapping errors made by genome aligners and to make read alignments more consistent in regions that contain indels. It follows the older GATK > v.4 best practice recommendation. It seems that this step is redundant, at least for RADseq data, but we kept it in our pipeline, similarly as we did in Záveská et al. (2019), or as applied in Brandrud et al. (2020). Then, ref_map.pl from Stacks was applied to the mapped bam files with the requirement of a minimum coverage to identify an allele (−m) of 4×. The programme Populations implemented in Stacks was used to export selected loci. Filtering on (i) number of heterozygous genotypes per locus, maximum observed heterozygosity (–max_obs_het) 0.65 to avoid combining paralogs within the same RAD locus, and (ii) missing data for a minimum of 20 out of 63 populations present with at least 20% of individuals from each population (−p 20 and −r 0.2 flags) were applied to all the datasets. For the first dataset, later used for ML phylogenetic reconstruction, multiple SNPs per locus were exported into full sequence phylip format using flags ‘—phylip,’ ‘–phylip-var,’ and ‘–phylip-var-all.’ For Bayesian clustering, a set of RADseq loci was exported into structure format using the –structure flag, and the –write-single-snp flag was used to select only a single (first) SNP per fragment to minimise the chance of selecting linked loci. Finally, for species tree reconstruction *via* SNAPP and for preparation of site frequency spectra (SFS) for demographic modelling, we selected two to 23 individuals with lowest proportion of missing data from each fast STRUCTURE group (see below). For those individuals, we exported an SNP per locus in vcf format using the programme Populations using the –vcf flag and further filtered using the VCFtools 0.1.15 (Danecek et al., 2011) for a maximum of 40% of missing data and a minimum depth of coverage of 10×.

### RADseq: Exploratory Analyses of SNP Data

To infer phylogenetic relationships among all 180 individuals of *A. saxatilis* and one outgroup sample of *O. argentea*, we computed a maximum likelihood (ML) phylogeny based on full sequences of 2,645 concatenated RADSeq loci using the RAxML 8.2.8 (Stamatakis, 2014) with random starting trees and the GTR + G nucleotide substitution model. The best-scoring ML tree was bootstrapped with the frequency-based stopping criterion (Pattengale et al., 2010).

For 180 individuals of *A. saxatilis*, the optimal grouping of populations was determined using the Bayesian clustering software for large SNP datasets and the fastSTRUCTURE v1.0 (Raj et al., 2014), and for each *K* from 1 to 10 with provided script structure.py using a simple prior. The optimal value of K was determined by the chooseK.py script packaged with the fastSTRUCTURE. The fastSTRUCTURE was used for the entire dataset and then for the three subsets representing three main groups inferred by the analysis of the entire dataset. Based on the same dataset, further filtered for maximum of 30% missing data, we plotted genetic distances among individuals by principal coordinate analysis (PCoA) calculated with the R package Adegenet 2.1.0 (Jombart, 2008; Jombart and Ahmed, 2011) based on Euclidean distances. To account for potential presence of hybridisation within the entire data set, we used the SplitsTree 4.12.6 (Huson and Bryant, 2006) to create a the NeighborNet based on Hamming distances (Hamming, 1950). The programme Populations in Stacks was used to estimate the number of private alleles and nucleotide diversity (π) per population.

To estimate divergence times, we used the species tree method SNAPP 1.5.1 (Bryant et al., 2012) in the BEAST 2.6.4 (Bouckaert et al., 2014) as described in Stange et al. (2018). We used a reduced data set containing 49 individuals proportionally sampled from five lineages identified by the RAxML and the fastSTRUCTURE, excluding the admixed east Balkan and North Macedonian populations (see Section “Results”). We constructed a new RAD data subset for these five lineages using the filtering parameters described above but requiring loci to be shared among all the 49 samples and further filtering of biallelic SNPs only. To scale the tree, we used a secondary calibration point obtained from the results of dating analysis based on the plastid data. In particular, the prior age of the root (i.e., diversification of *A. saxatilis*) was set to 2.2 Mya with a normally distributed SD of 0.9, which corresponds to the median age and 95% HPD interval of the corresponding node (see Section “Results”). To improve mixing and convergence of the model, we constrained the monophyly of two ingroup clades that were consistently resolved as monophyletic with strong support in RAxML analysis, in particular the Central European and East Balkan-Carpathian Group and the South Balkan-Apennine Group. We ran two independent MCMCs for 100,000 generations, discarding 30% of the generations as burn-in. Log and tree traces from the two runs were combined. We estimated the maximum clade credibility (MCC) tree from the posterior distribution using the TreeAnnotator 2.5.0.

### RADseq: Demographic Modelling

To investigate alternative divergence scenarios of the three geographically adjacent groups of *A. saxatilis* revealed by the fastSTRUCTURE analysis (the Core Central Balkan Subgroup, the East Balkan-Carpathian Group without admixed populations, and the Central European Group, see Section “Results”), we used the diffusion approximation method of dadi to analyse two-dimensional (2D-JSFS) site frequency spectra (Gutenkunst et al., 2009). We used an established 2D analysis pipeline (Portik et al., 2017; Charles et al., 2018) and adapted publicly available python scripts^5^ that define 2D models, perform model fitting, and execute plotting functions. As input data, we used a two-dimensional joint site frequency spectrum prepared *via* conversion of a vcf file to a folded SFS that included down-projection of the sampling size to maximise the number of sampled individuals while minimising levels of missing data for downstream multi-population comparisons and was performed using the easySFS tool^6^. We excluded all admixed individuals. The down-projection and filtering resulted in the following allele numbers: Core Central Balkan Subgroup, 24 alleles; East Balkan-Carpathian Group, 16 alleles; Central European Group, 18 alleles.

We applied a 2D analysis pipeline for pairwise comparison of two neighbouring groups of populations, i.e., the Core Central Balkan vs. the East Balkan-Carpathian Group and the East Balkan-Carpathian vs. the Central European Group, respectively. We tested whether one of the groups is a result of postglacial expansion from the other group, or if both groups survived at least the LGM in separate refugia. Potential scenarios for two groups, thus, represent events of (1) old (preglacial) vicariance, (2) old (preglacial) founder event, and (3) recent (postglacial) founder event as depicted in Supplementary Figure 2A *via* particular models (Supplementary Figures 2B, 3). Under the last two scenarios, founded groups are supposed to have experienced at least one bottleneck reflecting decrease in effective population size during the LGM followed by population expansion during the Holocene. However, detection of exponential growth in the last ten thousand years is challenging especially for organisms with long generation time (>10 years; Adams and Hudson, 2004; Elleouet and Aitken, 2018). Therefore, we chose to test simplified models, including either (i) no change in population size since the split, (ii) early stage of population expansion followed by constant population size, or (iii) continuously expanding population size (Supplementary Figure 2B). In a demographic context, we refer to genetic groups identified by the fastSTRUCTURE analyses as “populations” in accordance with recent studies (e.g., Portik et al., 2017; Charles et al., 2018).

To be able to differentiate the scenarios of a pre-glacial vs. a post-glacial split of the populations, we dated the splits between the groups using SNAPP (see above). For both pairwise comparisons, we tested eight island demographic models (‘island’ referring to the derived, recently founded population in contrast to the ancestral ‘mainland’ population; we use the more intuitive terms ‘derived population’ and ‘ancestral founding population’ in the following). These eight models represent the three main hypotheses and modifications with ancestral or recent migration included to explain additional features of the 2D-JSFS (Supplementary Figure 3). Models representing an old vicariance and a recent founder event were taken from Charles et al. (2018), and models of old founder events were designed in Záveská et al. (2021). For models in both the vicariance and founder event categories, we followed Charles et al. (2018) and included a variable “s” that defines the fraction of the ancestral population (nuA) founding each daughter population, where nuA*s represents the derived population and nuA*(1 − *s*) represents the ancestral founding population. We enforced an upper limit of 0.5 for *s*. In the optimization of the models, we followed Záveská et al. (2021). The optimised models were compared using Akaike information criterion (AIC), and the replicate with highest likelihood for each model was used to calculate AIC scores, ΔAIC scores and Akaike weights (ωi) (Burnham and Anderson, 2002). We did not transform parameters into biologically meaningful estimates, because our primary aim was to perform model selection.

### Species Distribution Modelling

To infer the potential present and past distribution of *A. saxatilis*, distribution models were generated using different modelling algorithms implemented in the *biomod2* R package (Thuiller, 2003; Thuiller et al., 2009, 2016) following an ensemble forecasting approach. Occurrence data were obtained through extensive fieldwork in a period that ranged from 2005 to 2019. Localities within a distance of 1 km^2^ were removed using the *spThin* R package (Aiello-Lammens et al., 2015) to account for spatial autocorrelation. The final dataset covering the whole geographic range of *A. saxatilis* consisted of 95 independent records, whereas the two subsets corresponding to two main genetic groups (see Section “Results” and Supplementary Figure 1) for which a sufficient number of geographic records was available included 38 (the South Balkan-Apennine Group) and 43 (the remainder of the area) records.

Eight climatic variables were selected based on their ecological relevance for *A. saxatilis* (Supplementary Table 3) and taken at a resolution of 30 arc-s (∼1 km) from the Chelsa Climate database (Karger et al., 2018). To account for potential multicollinearity between variables, variance inflation factor (VIF) and Pearson correlation coefficient were computed using the *usdm* R package (Naimi et al., 2014). Subsequently, three variables having a VIF factor greater than 10 or Pearson correlation coefficient (*r*) higher than 0.8 were excluded. The remaining five variables (temperature seasonality, bio04; mean daily mean air temperatures of the warmest quarter, bio10; precipitation amount of the wettest month, bio13; mean monthly precipitation amount of the driest quarter, bio17; mean monthly precipitation amount of the warmest quarter, bio18) were used for present and past species distribution modelling (SDM) of the total dataset as well as both subsets.

Projection of environmental space during the LGM period (approximately 22,000 years ago) was projected with the same set of variables available in the Chelsa climate PMIP3 dataset. Variables were calibrated according to seven different Paleoclimate Modelling Intercomparison Project (PMIP3) global circulation models (GCMs): NCAR-CCSM4, MRI-CGCM3, MPI-ESM-P, MIROC-ESM, CESS-FGOALS-g2, IPSL-CM5A-LR, and CNRM-CM5.

Species distribution modelling (SDM) was performed in *biomod2* by ensemble modelling. This method is based on the idea of “ensemble forecasting,” a method in which many models are developed using different statistical techniques and later combined in one final ensemble model that is the best possible combination of all developed models (Araújo and New, 2007). Species distribution models for each group were built using five modelling algorithms: Maximum Entropy (Maxent), Generalised Linear Model (GLM), Generalised Additive Model (GAM), Boosted Regression Trees (GBM), and Random Forest (RF). For each group and each algorithm, we ran 10 different models by cross-validation using a 70:30 data split, developing in total 50 models. Model performance was assessed based on the AUC value (“area under the receiver operating characteristic curve”) in two steps. In the first step, the median AUC value of all the 50 models was calculated, and in the second step, the ensemble modelling process was run again but only for models whose AUC value was higher than the calculated median value. In this manner, we selected only models that performed best across all runs and all modelling techniques. The ensemble model calculated in step two was the final model that was used in ensemble forecasting during the LGM period but for each GCM separately. To obtain the final habitat suitability model for LGM, we calculated the average pixel value across all GCMs.

## Results

### Plastid Sequence Phylogenies and Divergence Time Estimation

**FIGURE 1:**
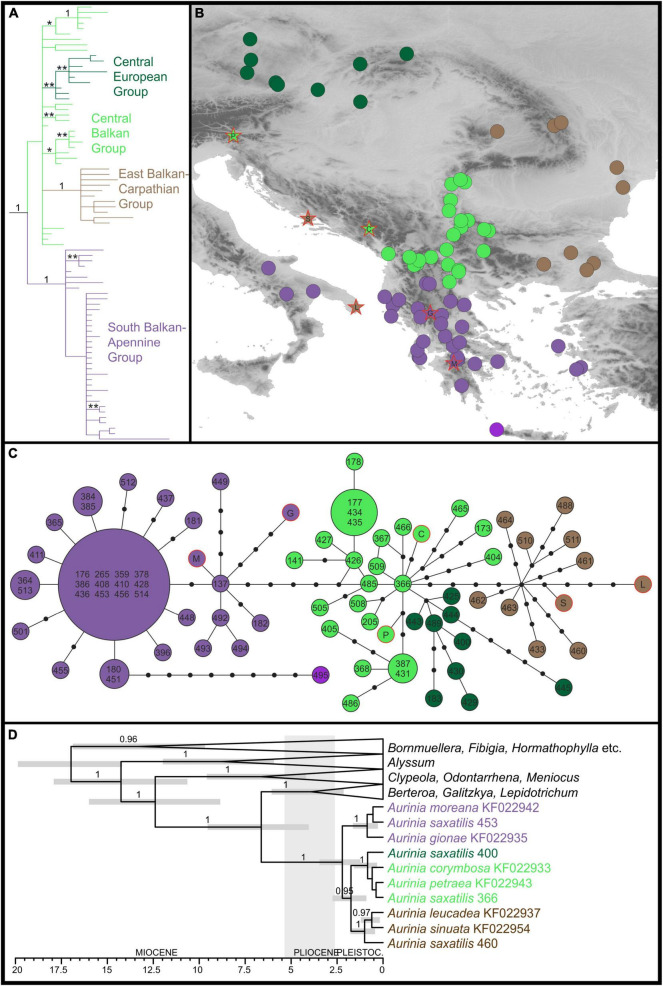
Phylogenetic relationships inferred by plastid *ndh*F sequences of *Aurinia saxatilis* and related species. The colour codes indicate the main groups. **(A)** Simplified Bayesian consensus phylogram of the ingroup sequences (as shown in Supplementary Figure 4); numbers/asterisks above branches are PP values: *0.90–0.94; **0.95–0.99. **(B)** Geographic distribution of the main clade/haplotype groups inferred by the Bayesian analyses **(A)** and the statistical parsimony haplotype network **(C)**. **(D)** Simplified Bayesian chronogram indicating the time of divergences in *Aurinia* and in relation to related genera (for details, see Supplementary Figure 5). Population identifiers in **(C,D)** correspond to Supplementary Table 1 and Supplementary Figure 1.

The plastid sequences of 75 *A. saxatilis* were 1,917 to 1,919 nucleotides long, after the exclusion of a section of ca. 70 bp between the first fragment (between primers 5F and 989R) and the second fragment (between primers 989F and 2100R). After the addition of six closely related *Aurinia* taxa and four outgroup sequences, the final alignment was 1,919-nucleotide long. Of 155 variable characters, 75 (3.9% of all the characters) were parsimony informative. A total of 2,499 best trees found in the parsimony analysis had a score of 188, and consistency index was 0.90 (0.82 after exclusion of uninformative characters). Bayesian and parsimony analyses resulted in largely congruent trees (Figure 1A and Supplementary Figure 4). *Aurinia* was resolved as monophyletic (MPB 100%, posterior probabilities, PP 1). In *Aurinia*, phylogenetic relationships were geography-correlated (Figure 1B) and several clades with good support (MPB > 59% and PP > 0.94) were resolved. One evolutionary lineage (MPB 77%, PP 1; violet in Figure 1), named hereafter the South Balkan-Apennine Group, included all accessions of *A. saxatilis* from the southern Balkan Peninsula (including the Aegean islands) and Italy, and the sympatric Greek endemic *Aurinia gionae* and *Aurinia moreana*. The second evolutionary lineage (MPB 90%, PP 1; brown in Figure 1), named hereafter the East Balkan-Carpathian Group, included all accessions of *A. saxatilis* from the eastern Balkan Peninsula, eastern Carpathians, and Apuseni Mts, and of allopatric *Aurinia leucadea* and *Aurinia sinuata*, both endemic to the Adriatic Basin. The third lineage (MPB 60%, PP 0.96; dark green in Figure 1), named hereafter the Central European Group, included all accessions of *A. saxatilis* from the northern Pannonian Basin, Western Carpathians, and Moravia and Bohemia. All other accessions of *A. saxatilis* distributed in the central Balkan Peninsula (light green in Figure 1) are named hereafter the Central Balkan Group; they were in basal polytomy (considering the support of MPB 55% and PP 0.52 as non-relevant) including central and southern Balkan *Aurinia corymbosa* and disjunctly distributed southeast Alpine and south Carpathian *Aurinia petraea*. In this group, the northwestern populations 368, 387, 405, 431, and 486 formed a clade with MPB 86% and PP 1, and the easternmost populations 177, 178, 434, and 435 formed a clade with MPB 63% and PP 0.95. In addition, some pairs of mostly geographically allied accessions had MPB > 59% and PP > 0.94 (see Supplementary Figure 4 for details).

The phylogenetic relationships revealed in the trees were also reflected in the TCS haplotype network (Figure 1C): the Central Balkan Group was six mutational steps both from the South Balkan-Apennine Group and the East Balkan-Carpathian Group, whereas the Central European Group was only two mutational steps away from the first group. The South Balkan-Apennine Group was relatively uniform, with several populations sharing the same haplotype, with the most divergent being mostly those from the Aegean Basin, especially Crete (population 495). In all the other groups, haplotype diversity was higher, and most of the populations had their own haplotypes, and some of them were rather divergent (e.g., 173, 404, 405, 445, 488, and 460).

The phylogenetic relationships and inferred divergence times within the tribe Alysseae (Figure 1D and Supplementary Figure 5) corresponded to those inferred in Huang et al. (2020). *Aurinia* was monophyletic (PP 1) and originated 6.6 Mya (HPD 4–9.5), but started to diversify only 2.2 Mya (HPD 1.2–3.5) and continued to diversify throughout the Pleistocene. In *Aurinia*, the inferred relationships corresponded to the main groups inferred by phylogenetic analyses of the complete *Aurinia* dataset (see above), with the exception that a sister relationship between the South Balkan-Apennine Group (including *A. gionae* and *A. moreana*; PP 1) and all the other groups (PP 0.95) was inferred. In the latter clade, one lineage (PP 1) corresponded to the East Balkan-Carpathian Group (including *A. leucadea* and *A. sinuata*), and the other (PP 1) to the combined Central Balkan and Central European Groups.

### RADseq Phylogenetic Relationships

#### Population Structure and Phylogenetic Relationships

The average number of high-quality reads per sample retained after demultiplexing and quality filtering was 0.68 million (*SD* = 0.21). The data have been deposited in the NCBI Short Archive (BioProject PRJNA761287, accessions number SRR15735925–SRR15736104).

**FIGURE 2:**
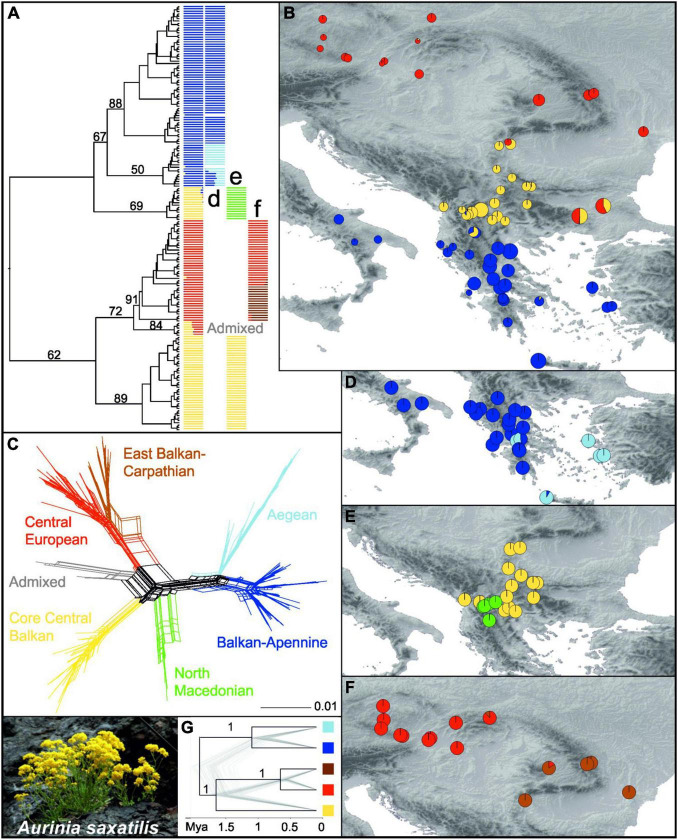
Genetic structure in *Aurinia saxatilis* (photo by I. Rešetnik in the bottom left corner) based on restriction site-associated DNA sequencing (RADseq). **(A)** Simplified maximum likelihood tree as shown in Supplementary Figure 6, without outgroup and with bootstrap support of major clades above branches. The tree is complemented with barplot representations of the three main fastSTRUCTURE groups shown in **(B)** and their subgroups shown in **(D–F)**. In **(B)**, sizes of the pie charts reflect the number of private alleles in each population. **(C)** NeighborNet based on Hamming distances. **(G)** Simplified SNAPP analysis species tree with bootstrap support of major clades above branches, as shown in Supplementary Figure 9.

A RAxML phylogenetic tree based on 27,791 SNPs (Supplementary Table 4) that was rooted using *O. argentea* resolved *A. saxatilis* as monophyletic (Figure 2A and Supplementary Figure 6). In *A. saxatilis*, four main clades with bootstrap support (BS) > 65% were identified. With the exception of the clade having BS 69%, they largely corresponded to the three main groups inferred by Bayesian population clustering based on 7,005 unlinked SNPs (Supplementary Table 4) using fastSTRUCTURE (colour codes in the first column in Figure 2A). The groups were allopatrically distributed (Figure 2B). With the exception of the strongly admixed populations 433 and 488 between the red and the yellow clusters, and 428 between the blue and the yellow clusters, as well as the population 466 that was included in the red cluster, the RADseq clusters corresponded to the groups inferred with plastid sequences (see above; Figure 1), but the Central European Group and the East Balkan-Carpathian Group were united in the same cluster; therefore, for simplicity, we apply the same names of the groups as used for the plastid dataset. The South Balkan-Apennine Group (BS 67% in the RAxML tree) was divided into a subcluster composed of the Balkan and Apennine mainland populations (dark blue; Balkan-Apennine Subgroup), and a subcluster of populations from Crete and the Aegean islands (light blue; Aegean Subgroup; Figure 2D). The two subgroups were clearly divergent in NeighborNet (Figure 2C) and had BS 88 and 50% in the RAxML tree, respectively (Figure 2A and Supplementary Figure 6). The Central Balkan Group inferred with fastSTRUCTURE (yellow in Figure 2B) was split into two divergent lineages in the RAxML tree, with one having BS 89% and the other having BS 69% (Figure 2A and Supplementary Figure 6), which corresponded to two fastSTRUCTURE subclusters (Figure 2E), also clearly divergent in NeighborNet (Figure 2C): the North Macedonian Subgroup (green in Figure 2E) and the Core Central Balkan Subgroup (yellow in Figure 2E).

The combined East Balkan-Carpathian and Central European Groups (red in Figure 2B; BS 72% in the RAxML tree) included two strongly admixed populations, 488 and 433, from the eastern Balkan Peninsula that formed the earliest diverging lineage (BS 84%) within the group in the RAxML tree and were positioned intermediate between the Core Central Balkan Subgroup (yellow) and the remainder of the combined East Balkan-Carpathian and Central European Group (red–brown) in NeighbourNet (grey; Figure 2C and Supplementary Figure 6). These two combined groups were least divergent in NeighbourNet but divided by a fastSTRUCTURE analysis (brown and red; Figure 2F).

The scatterplot based on the principal coordinate analysis (PCoA) of 2,279 unlinked SNPs (Supplementary Figure 7) was highly congruent with the results of the RAxML, NeighbourNet, and fastSTRUCTURE analyses and revealed similar groupings of populations. Distances among the Core Central European Subgroup (yellow), combined East Balkan-Carpathian and Central European Groups (red-brown), and South Balkan-Apennine Group (blue) were similar, as revealed by the principal coordinates 1 and 2, while the admixed populations 433 and 488 from the eastern Balkan Peninsula and those of the North Macedonian Subgroup were intermediate among them; the latter were strongly divergent along the principal coordinate 3. The first three principal coordinates explained over 39% of the variation.

Although the total number of (both variable and invariant) sites recovered was comparable among the six subgroups (Table 1), the number of private alleles and nucleotide diversity (π) per population differed significantly among some of them (Figure 2B and Supplementary Figure 8). The lowest amounts of private alleles and lowest π values were observed in the Central European Group. The highest amount of private alleles was observed in the Aegean Subgroup, and highest π values were observed in the North Macedonian Subgroup (Table 1 and Supplementary Figure 8).

**TABLE 1:** Descriptors of the groups of populations of *Aurinia saxatilis* identified with restriction-site associated DNA sequencing (RADseq) data.

	Sites recovered	% polymorphic loci	Expected heterozygosity	Nucleotide diversity (π)	Private alleles
Central European Group	406119	3.4	0.0001	0.0001	31.3
East Balkan-Carpathian Group	361674.2	4.3	0.0001	0.0002	55.6
Core Central Balkan Subgroup	401131.1	4.8	0.0002	0.0003	48.3
North Macedonian Subgroup	425340.6	6.7	0.0003	0.0004	72.8
Balkan-Apennine Subgroup	421975.9	6.4	0.0002	0.0003	66.7
Aegean Subgroup	358335.2	4.5	0.0001	0.0002	81.7

*The values are averaged over populations within each group.*

#### Time-Calibrated Nuclear Data-Based Phylogeny

The species tree analysis SNAPP conducted for inference of a time-calibrated phylogeny was based on 1,914 unlinked SNPs and resulted in same relationships among the main lineages as in the RAxML tree but with higher support (PP 1) for all nodes (Figure 2G and Supplementary Figure 9). The onset of diversification of *A. saxatilis* was estimated at 1.9 Mya (95% HPD 0.6–2.9 Mya), followed by divergence between the Core Central Balkan Subgroup and the combined Central European and East Balkan-Carpathian Groups at 1.6 Mya (95% HPD 0.5–2.5 Mya). The Balkan-Apennine and Aegean subgroups diverged 1 Mya (95% HPD 0.3–1.6 Mya), followed by the divergence between the Central European Group and the East Balkan-Carpathian Group 0.6 Mya (95% HPD 0.2–0.9 Mya).

#### Demographic History

**FIGURE 3:**
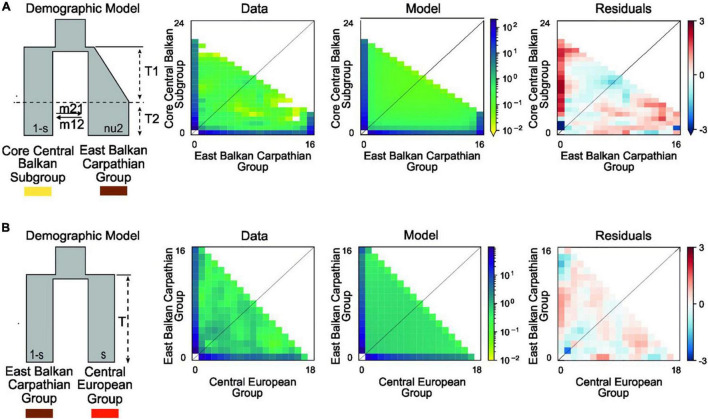
Best-fitting demographic models for various phylogeographic groups of *Aurinia saxatilis* using a two-dimensional joint site frequency spectrum (2D-JSFS) between population sets including **(A)** the Core Central Balkan Subgroup (*n* = 24) and the East Balkan-Carpathian Group (*n* = 16) and **(B)** the East Balkan-Carpathian Group (*n* = 16) and the Central European Group (*n* = 18). A visual representation of the best-fit model is depicted, along with comparisons of the 2D-JSFS for data, model, and resulting residuals. For both comparisons, the best-fit model represents the scenario where the split is theoretically pre-glacial (see Supplementary Figure 2 and Section “Introduction”). Additional models and parameter values are provided in Table 2 and Supplementary Table 5.

Detailed results of our demographic modelling are given in Table 2, Supplementary Table 5, and Figure 3. In both comparisons, the models of old divergence events fitted better to our data than the model of a recent founder event (Supplementary Table 5).

**TABLE 2:** Two best demographic models and unscaled parameter values for pairwise population comparisons.

population 1 vs. population 2	Model	Log-likelihood	ωi	Theta	nuA	nu1	nu2	T	T1	T2	*s*	m12	m21
Core Central Balkan Subgroup vs. East Balkan-Carpathian Group	founder_sec_contact_asym_two_epoch	−238.3	0.81	331.45	0.43	10.01	0.36		0.19	0.5	0.1	0.23	0.06
	vic_sec_contact_asym_mig	−240.4	0.10	171.1	1.4	0.03	1.05		0.43	0.68	0.4	0.09	0.05
East Balkan-Carpathian Group vs. Central European Group	vic_no_mig	−177.32	0.427	96.62	1.54	1.64	14.9	0.4			0.3		
	founder_anc_asym_two_epoch	−175.33	0.156	458.67	0.2	7.24	0.12		0.17	0.02	0.01	1.77	6.12

*Detailed results of all the models tested are shown in Supplementary Table 5. Abbreviations are as follows: founder_sec_contact_asym_two_epoch, old founder event with exponential size growth followed by asymmetric migration in the second epoch; vic_sec_contact_asym_mig, vicariance with no migration followed by asymmetric migration in the second epoch; vic_no_mig, vicariance with no migration; founder_anc_asym_two_epoch, old founder event with exponential size growth and continuous asymmetric migration followed by isolation; ω_i_, Akaike weight; Theta [4N_ref_μL, where L is the total length of the sequenced region from which single nucleotide polymorphisms (SNPs) were ascertained], effective mutation rate of the reference population (here corresponds to the ancestral population); nuA; effective population size of the ancestral population; nu1 and nu2, effective population sizes of populations 1 and 2, respectively, under the constant population size model; T, unscaled time of population split; T1, unscaled time between population split and ancient migration; T2, unscaled time between ancient migration and the present; s, fraction of the nuA running into population 2; m12, migration rate from population 2 to population 1; m21, migration rate from population 1 to population 2.*

In comparison of the Core Central Balkan Subgroup and the East Balkan-Carpathian Group, we found a strong support (81% of the total model weight) for an old founder event with exponential size growth followed by a continuous asymmetric migration in the second epoch (ΔAIC = 4.18, ω_i_ = 0.81; Figure 3A, Table 2, and Supplementary Table 5). The proportion of ancestral founder population in the Carpathian Subgroup was estimated to be 0.1, and gene flow was inferred to be stronger from the East Balkan-Carpathian Group to the Core Central Balkan Subgroup (*m*_12_ = 0.23, *m*_21_ = 0.06; Table 2). The second best model (ω_i_ = 0.1) represents a similar scenario where the old founder event was replaced by a vicariance, while there was no migration in the first epoch, followed by an asymmetric migration in the second epoch.

In comparisons of the East Balkan-Carpathian and the Central European Groups, we performed model testing, so both groups acted either as ancestral population in founder event models or major fraction of the ancestral population in case of vicariance models. The best fitting model (ΔAIC = 2.02, ω_i_ = 0.42; Figure 3B) was a vicariance model with no migration (Table 2 and Supplementary Table 5) where the East Balkan-Carpathian Group acted as a major fraction of the ancestral population (1 − s = 0.7). The second best model (ΔAIC = 2.3, ω_i_ = 0.15; Supplementary Table 5) suggested an old founder event of the Central European Group from the East Balkan-Carpathian Group, followed by an exponential size growth of the Central European Group and continuous asymmetric migration ending with isolation.

### Species Distribution Modelling

**FIGURE 4:**
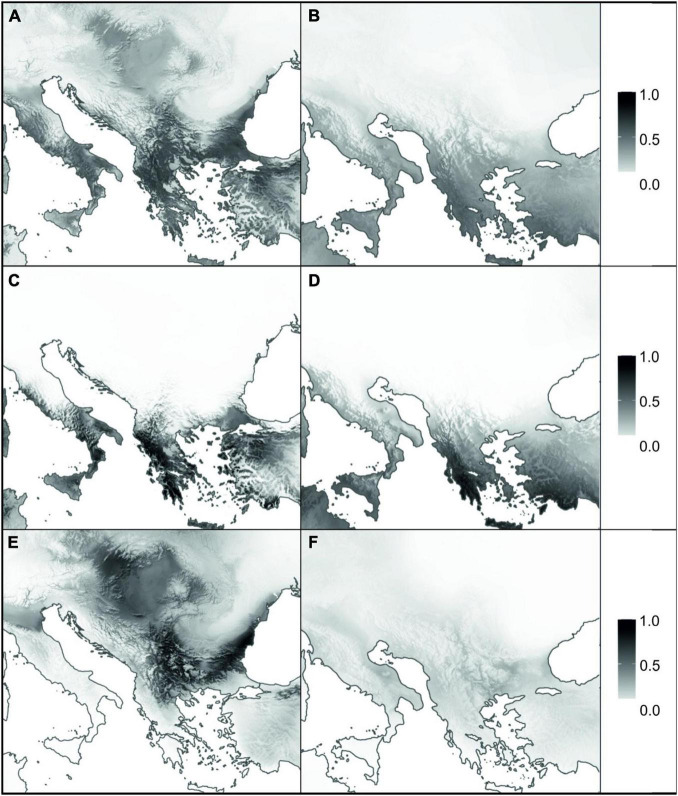
Species distribution models for *Aurinia saxatilis* under current **(A,C,E)** and Last Glacial Maximum **(B,D,F)** climatic conditions. Panels **(A,B)** represent the models for complete distribution of *A. saxatilis*, whereas **(C–F)** are models for two distinct evolutionary lineages of *A. saxatilis*, namely the South Balkan-Apennine Group and the rest of the distribution, respectively (marked with blue and red symbols in Supplementary Figure 1). Grey shading corresponds to habitat suitability, ranging from 0 (white: no suitability) to 1 (black: maximum suitability).

Mean AUC scores showed that the performance of all the models using current climate data was excellent (AUC > 0.95 in all cases; Supplementary Table 6). Moreover, sensitivity and specificity scores were always above 83, confirming the well-balanced performance of the developed models for current conditions. The present modelled habitat suitability agreed with the actual distribution of *A. saxatilis*, but some additional suitable areas with no known occurrences were indicated (Figure 4A). Additional regions identified as climatically highly suitable were the Black Sea coast and southerly adjacent regions of Asia Minor as well as the northwestern Apennine Peninsula and Sicily, and, to a lesser extent, the Adriatic Sea coastal area. SDMs hindcasted to the LGM revealed the southern parts of the Apennine and the Balkan Peninsula (including the Aegean islands and Crete) as well as Asia Minor as main areas of high suitability (Figure 4B).

Separate analyses of the South Balkan-Apennine Group indicated a long-term climatic stability and comparable area suitability for *A. saxatilis* at present and during the LGM (Figures 4C,D). On the other hand, the Central Balkan, East Balkan-Carpathian, and Central European Groups were predicted to have undergone substantial range contraction during the LGM compared to the present; there is limited support for potential refugia in southeastern Balkan-Pontic region and adjacent southern margins of the Carpathians (Figures 4E,F).

## Discussion

Our phylogenetic reconstructions coupled with species distribution modelling revealed that *A. saxatilis* had a long and dynamic history characterised by both vicariance and dispersal throughout Pleistocene, resulting in allo- to peripatrically distributed phylogeographic lineages. Both nuclear and plastid data suggested long-term isolation of the southern populations spanning the Eastern Mediterranean, from the southernmost Balkan Peninsula to the Aegean Basin and the southern Apennine Peninsula, with relative stability through time revealed by SDM. On the other hand, more northern inland populations experienced a turbulent history likely because of more severe impacts of Pleistocene climatic oscillations, triggering range contractions and secondary contacts with gene flow during range expansions. The results are more complex genetic patterns, which are partly incongruent between the plastid and nuclear data, although the main phylogeographic lineages match between the datasets. Our study, thus, corroborates the importance of the Eastern Mediterranean as a refugial area, enabling persistence of several lineages through time, but to a lesser extent its importance as a cradle of diversification (Mansion et al., 2009; Barres et al., 2013). In *A. saxatilis*, the cradle of diversification was the central Balkan Peninsula from where, like in many other plant species (e.g., Magri et al., 2006; Bardy et al., 2010; Ronikier, 2011; Đurović et al., 2017; Kuzmanović et al., 2021), the Carpathians and Central Europe were colonised.

The molecular dating based on plastid sequences placed the origin of *Aurinia* in the Miocene–Pliocene boundary 6.6 Mya (95% HPD 4–9.5), with the onset of diversification of *A. saxatilis* dated to early Pleistocene 2.2 Mya (95% HPD 1.2–3.5; Figure 1D), or slightly later, based on dating analyses of the RADseq data (1.9 Mya, 95% HPD 0.6–2.9; Figure 2G). The diversification continued throughout the Pleistocene, and likely all main lineages diverged before the LGM, as the most recent divergence, i.e., the split between the Central European and the East Balkan-Carpathian Groups was dated to be 0.6 Mya (95% HPD 0.2–0.9). Various other temperate species exhibit deep divergences dating back to the mid Pleistocene, indicating that lineages can remain distinct during repeated glacial and interglacial intervals (e.g., Bardy et al., 2010; Záveská et al., 2021).

### Long Term Persistence and Stability in the Eastern Mediterranean: Mountain Ridges and Sea Shaping Genetic Structure

Both nuclear-derived RADseq data and maternally inherited plastid sequences revealed a clear distinction of the southern populations of *A. saxatilis* from the southernmost Balkan Peninsula and the adjacent Aegean Basin, with a disjunction in the southern Apennine Peninsula (i.e., the South Balkan-Apennine Group). This area is characterised by a Mediterranean climate (Peel et al., 2007) and long-term climatic stability inferred by SDM, as areas suitable for *A. saxatilis* in this part of Europe did not differ considerably between the present and the LGM (Figures 4A–D), suggesting that the populations could persist in their locations throughout the Pleistocene and Holocene. A continuous persistent distribution in the mainland area with possible gene flow among populations resulted in a relatively uniform genetic structure, as a single haplotype is shared across multiple populations (Figure 1C) and relationships in the RADseq tree are poorly resolved in this group (Figure 2 and Supplementary Figure 6). Outstanding in this respect is the mountainous Pindus range in central Greece, where populations harbour a larger number of private alleles (Figure 2B) and some populations also possess unique haplotypes (Figure 1C), suggesting local and weakly interconnected gene pools. As higher elevations were more affected by Pleistocene glaciations and the snow line on the Balkan Peninsula during the LGM was approximately 1,000 m lower than today (Turrill, 1929; Horvat et al., 1974), vertical migrations accompanied by range contractions and displacements leading to bottlenecks and hybridisation likely contributed to increased genetic peculiarity in this region. As *A. saxatilis* is typically found at mid-altitudes in deep valleys up to 1,000 m, higher mountain ridges can still act as a barrier to gene flow.

Contrary to the high genetic diversity in the mountainous areas over small distances, disjunct distant populations in the Apennine Peninsula are genetically depauperate and highly similar to the western Greek populations (Figures 1, 2), which suggest their recent dispersal across the Adriatic Sea. In the RAxML tree of the RADseq data (Supplementary Figure 6), they diverged from the southern Balkan populations close to the tips of the tree, and two Apennine populations share the same haplotype with the southern Balkan populations (Figure 1C). *Aurinia saxatilis* is thus another example of recent, i.e., Pleistocene, *trans*-Adriatic dispersal and colonisation of the Apennine Peninsula (Frajman and Schönswetter, 2017; Falch et al., 2019; Garcia-Jacas et al., 2019; Janković et al., 2019) and corroborates the important role that the Balkans had not only for Central European diversity but also for the high diversity of the Apennine Peninsula.

The east- and southward expansion from mainland Greece to the Aegean archipelago and Crete happened much earlier, as the Aegean RADseq lineage, including a population from Attica, is the most early divergent in the South Balkan-Apennine Group (Figure 2A and Supplementary Figure 6), suggesting long isolation from the mainland populations and most likely recent migration back to Attica. As Crete was separated from the mainland since the Messinian Salinity Crisis (5.96–5.33 Mya; Poulakakis et al., 2015), i.e., much before the onset of diversification in *A. saxatilis*, the Crete and Aegean islands were likely colonised *via* long-distance dispersal of light-winged seeds. This migration was facilitated through land bridges during Pleistocene glaciations, enabling stepping-stone migrations. The expansion across the Aegean Basin was much more recent than the origin of the Aegean group, a scenario supported by a long branch and late diversification of this lineage in the RAxML tree (Supplementary Figure 6). However, whether the species persisted in isolation from the southern Balkan populations on one (or few) of the Aegean islands or in the westernmost Asia Minor, where *A. saxatilis* also occurs (Dudley, 1964), is impossible to answer because of our poor sampling in the Aegean basin. The colonisation of islands was likely connected with strong bottlenecks accompanied by genetic drift, as the Cretan population also harbours a high number of private alleles (Figure 2B) and a very divergent plastid haplotype (Figure 1C). The sea in the Aegeus, thus, likely had a similar effect on genetic structure as the high mountain peaks and ridges of the Pindus.

### Turbulent Pleistocene History: From the Central Balkan Peninsula Over Carpathians to Central Europe

Contrary to the southern Balkan Peninsula that had high climatic stability through time, more northern populations of *A. saxatilis*, nowadays ranging to Central Europe, experienced a more turbulent history, as during the LGM (and probably during other glacials), climatically suitable areas decreased considerably (Figure 4F), triggering local extinctions and migrations. The main split between the south Balkan-Aegean populations and all other populations corresponds well with the boundary of the southern oro-Mediterranean and the northern temperate-continental climate (Horvat et al., 1974; Ronikier, 2011). Locally glaciated high mountain ranges of northern Greece could have acted and still act as a strong climatic barrier and a barrier to migration and gene flow of plants that are typically found at mid altitudes (up to 1,000 m) or below, as in the case of *A. saxatilis*, the *Veronica chamaedrys* group (Bardy et al., 2010), *Edraianthus graminifolius* (Surina et al., 2014), and *Campanula versicolor* (Janković et al., 2019).

The divergence among the main lineages of *A. saxatilis* spanning from the central and eastern Balkan Peninsula over the Carpathians to Central Europe happened soon after the main genetic split discussed above. It is likely that climatic cooling and aridification that resulted in decrease of forested areas and increase in steppes (Svenning, 2003; Kirschner et al., 2020) enabled the rapid spread of *A. saxatilis* across southeast Europe in the early Pleistocene. This was followed by divergence in isolated refugia during glacial maxima, when climatically suitable areas diminished considerably as evidenced for LGM by SDM (Figures 4E,F). Smaller areas appropriate for species survival during the LGM (and likely other glacials) were more abundant in the central and eastern Balkans and southern margins of the Carpathians (Figure 4). Our demographic modelling (Figure 3) suggests that the east Balkan-Carpathian populations were derived from the central Balkan populations *via* an old founder event, which is consistent with the plastid data, where a long branch in the tree leads to the East Balkan-Carpathian Group from the polytomy of the Central Balkan populations (Figure 1). Colonisation of the Carpathians and the east Balkan-Pontic region from the central Balkan Peninsula was evidenced also in other plant groups (e.g., Frajman and Oxelman, 2007; Puşcaş et al., 2008; Csergö et al., 2009; Đurović et al., 2017).

Whereas the Central Balkan Group of *A. saxatilis* likely persisted glacial cycles *in situ* at lower altitudes, the East Balkan-Carpathian Group possibly had its refugium in the southern margin of the Carpathians and adjacent Pontic region. A similar genetic break, as inferred between the Central Balkan and East Balkan-Carpathian populations of *A. saxatilis*, is also seen in *Sesleria rigida* s.l. (Kuzmanović et al., 2013) and between vicariant sister species such as *Euphorbia niciciana* and *Euphorbia seguieriana* (Frajman et al., 2019) as well as *Campanula orbelica* and *Campanula alpina* (Ronikier and Zalewska-Gałosz, 2014), suggesting that several plants had glacial refugia divergent between the central Balkan Peninsula and the Carpathians.

Less clear is the origin of the Central European populations of *A. saxatilis* that diverged relatively late from the East Balkan-Carpathian Group, as suggested by the RADseq data (Figure 2), which best fit to a vicariant divergence (Figure 3), whereas the plastid data suggest an origin directly from the Central Balkan Group in a manner similar to that of the East Balkan-Carpathian Group (Figure 1). Independently of the origin of the Central European population, their high genomic similarity with the East Balkan-Carpathian populations and their late divergence indicated by the RADseq data (Figure 2G) suggest that they were connected throughout the majority of Pleistocene, and that they diverged before the LGM and, thus, likely had separate glacial refugia at least during the LGM. This is similar to the situation of *Cyclamen purpurascens* where one of the LGM refugia was suggested to be in the Tatra Mts (Slovák et al., 2012) and *Arabidopsis arenosa* for which Pannonian Basin and western Carpathians as northern refugia were postulated (Kolář et al., 2016). Enclaves of both coniferous and deciduous broadleaved trees, together with understory biota, existed in microclimatically favourable sites of central Europe (e.g., Magri et al., 2006; Kramp et al., 2009; Slovák et al., 2012), where *A. saxatilis* could have also survived the LGM, for example, in the Pannonian Basin where low climatic suitability was indicated by SDM (Figure 4).

As expected for a temperate species like *A. saxatilis*, its range increased in the face of climate warming in interglacials, and population groups isolated in glacial refugia came to secondary contacts, as evidenced by admixture patterns revealed by the RADseq data between the Central Balkan and the South Balkan-Apennine, as well as the Central Balkan and East Balkan-Carpathian Groups (Figure 2). Gene flow between the groups was evidenced not only by fastSTRUCTURE analyses but also by the intermediate position of some North Macedonian and eastern Balkan populations in NeighbourNet. Correspondingly, the high number of private alleles, high nucleotide diversity, and expected heterozygosity in the hybrid populations in North Macedonia and those in the eastern Balkans could be the result of the mixture of different genomic pools (Figure 2B, Table 1, and Supplementary Figure 8).

In summary, the central Balkan to central European populations of *A. saxatilis* had a turbulent demographic history highly influenced by Pleistocene climatic oscillations. They triggered bottlenecks and genetic drift during population contractions, and hybridisation during their expansions, resulting in complex genetic patterns, high plastid haplotype diversity and numerous unique haplotypes separated by multiple mutation steps (Figure 1) as well as slight incongruences between the plastid and genome-wide data. These processes contributed to high genetic diversity in the central parts of the Balkan Peninsula also in other plant groups (López-Vinyallonga et al., 2015; Đurović et al., 2017; Caković et al., 2021; Cetlová et al., 2021) and highlight this area not only as a cradle of lineage diversifications, but also as a source of lineage dispersals.

### Taxonomic Implications

Despite the fact that other *Aurinia* species are nested in different clades of *A. saxatilis* in our plastid tree, either within sympatrically distributed populations as in the case of *A. gionae* and *A. moreana* or with allopatric populations being hundreds of kilometres away as in the case of *A. corymbosa*, *A. leucadea*, *A. petraea*, and *A. sinuata* (Figure 1), the nuclear data resolved *A. saxatilis* as monophyletic (ITS data in Rešetnik et al., 2013, preliminary analyses of RADseq data including all *Aurinia* species, Rešetnik et al., unpublished). Reasons for this incongruence between the nuclear and the plastid data could be incomplete lineage sorting, but also hybridisation and chloroplast capture (Rieseberg and Soltis, 1991) in the case of sympatric species.

*Aurinia saxatilis* is morphologically strongly polymorphic, and three subspecies were recognised (Dudley, 1964; Persson, 1971; Akeroyd, 1993; Plazibat, 2009). However, the quantitative characters used in subspecies delimitation (size, shape and pubescence of leaves, size and shape of siliculae, and length of styles) vary considerably among populations (Rešetnik, personal observations). Interestingly, the main genetic break inferred by both plastid and nuclear data in our study among the central and eastern Balkan, Carpathian, and central European populations on one side and the southern Balkan-Apennine populations on the other correspond to *A. saxatilis* subsp. *saxatilis* and *A. saxatilis* subsp. *orientalis*, respectively. The main morphological characters distinguishing the two subspecies are related to the siliculae and rosette leaves: siliculae smaller and longer than wide with rounded apex, rosette leaves usually entire (*A. saxatilis* subsp. *saxatilis*), vs. siliculae larger and the same width and length or wider than longer, with emarginate or truncate apex, rosette leaves usually dentate to pinnatifid (*A. saxatilis* subsp. *orientalis*; Persson, 1971). Morphologically intermediate individuals occur mostly along the North Macedonian-Greek border (Persson, 1971), i.e., in the area where genetically admixed populations have been revealed by our study (Figure 2B).

The third subspecies, *A. saxatilis* subsp. *megalocarpa* is reported to be parapatric with *A. saxatilis* subsp. *orientalis*, with the former having larger quantitative characters of siliculae, style and seed wings (Dudley, 1964; Persson, 1971; Akeroyd, 1993). However, the measurements performed by Dudley (1964) and Persson (1971) are not congruent. They also partly disagree in distribution of *A. saxatilis* subsp. *megalocarpa*, for which Dudley (1964) indicated the Aegean islands and western Anatolia, whereas Persson (1971) also included southern Italy. The genetic divergence between the Balkan-Apennine Subgroup and the Aegean Subgroup in our RADseq data (Figure 3), thus, largely corresponds to Dudley’s (1964) delimitation. More detailed morphological analyses with dense geographic sampling are needed to test the morphological divergence between both subspecies and generate precise data on their distributions and, thus, validate the taxonomic recognition of *A. saxatilis* subsp. *megalocarpa*.

## Data Availability Statement

The datasets presented in this study can be found in online repositories: https://www.ncbi.nlm.nih.gov/genbank/, OK180986-OK181056, https://www.ncbi.nlm.nih.gov/, Bio-Project PRJNA761287, accessions number SRR15735925-SRR15736104.

## Author Contributions

IR conceived the study. IR and SB collected the majority of samples with additional samples provided by colleagues (listed in Supplementary Table 1). IR carried out the RADseq and PB plastid molecular laboratory work. IR and BF analysed the genetic data. EZ analysed the genomic data. MG produced the species distribution models. IR and BF wrote major parts of the manuscript, with exception of the parts about RADseq written by EZ. All authors read and edited the final version of the manuscript.

## Conflict of Interest

The authors declare that the research was conducted in the absence of any commercial or financial relationships that could be construed as a potential conflict of interest.

## Publisher’s Note

All claims expressed in this article are solely those of the authors and do not necessarily represent those of their affiliated organizations, or those of the publisher, the editors and the reviewers. Any product that may be evaluated in this article, or claim that may be made by its manufacturer, is not guaranteed or endorsed by the publisher.

## Footnotes

1
http://www.vbcf.ac.at/ngs/

2
https://www.geneious.com

3
https://github.com/wtsi-npg/illumina2bam

4
http://broadinstitute.github.io/picard

5
https://github.com/dportik/dadi_pipeline

6
https://github.com/isaacovercast/easySFS

## References

[B1] Adams A. M. Hudson R. R. (2004). Maximum-likelihood estimation of demographic parameters using the frequency spectrum of unlinked single nucleotide polymorphisms. *Genetics* 168 1699–1712. 10.1534/genetics.104.030171 15579718 PMC1448761

[B2] Aiello-Lammens M. E. Boria R. A. Radosavljevic A. Vilela B. Anderson R. P. (2015). spThin: an R package for spatial thinning of species occurrence records for use in ecological niche models. *Ecography* 38 541–545.

[B3] Akeroyd J. R. (1993). “*Aurinia* (L.) Desv,” in *Flora Europaea 1*, 2nd Edn, eds Tutin T. G. Heywood V. H. Burges N. A. Valentine D. H. Moore D. M. (Cambridge: Cambridge University Press), 369–371.

[B4] Araújo M. B. New M. (2007). Ensemble forecasting of species distributions. *Trends Ecol. Evol.* 22 42–47. 10.1016/j.tree.2006.09.010 17011070

[B5] Bardy K. E. Albach D. C. Schneeweiss G. M. Fischer M. A. Schönswetter P. (2010). Disentangling phylogeography, polyploid evolution and taxonomy of a woodland herb (*Veronica chamaedrys* group, Plantaginaceae s.l.) in southeastern Europe. *Mol. Phylogenet. Evol.* 57 771–786. 10.1016/j.ympev.2010.06.025 20603220 PMC2989448

[B6] Barres L. Sanmartín I. Anderson C. L. Susanna A. Buerki S. Galbany-Casals M. (2013). Reconstructing the evolution and biogeographic history of tribe Cardueae (Compositae). *Am. J. Bot.* 100 867–882. 10.3732/ajb.1200058 23624927

[B7] Bartha L. Sramkó G. Volkova P. A. Surina B. Ivanov A. L. Banciu H. L. (2015). Patterns of plastid DNA differentiation in *Erythronium* (Liliaceae) are consistent with allopatric lineage divergence in Europe across longitude and latitude. *Plant Syst. Evol.* 301 1747–1758. 10.1007/s00606-014-1190-x

[B8] Beilstein M. A. Al-Shehbaz I. A. Kellogg E. A. (2006). Brassicaceae phylogeny and trichome evolution. *Am. J. Bot.* 93 607–619. 10.3732/ajb.93.4.607 21646222

[B9] Bouckaert R. Heled J. Kühnert D. Vaughan T. Wu C.-H. Xie D. (2014). BEAST 2: a software platform for bayesian evolutionary analysis. *PLoS Comput. Biol.* 10:e1003537. 10.1371/journal.pcbi.1003537 24722319 PMC3985171

[B10] Brandrud M. K. Baar J. Lorenzo M. T. Athanasiadis A. Bateman R. M. Chase M. W. (2020). Phylogenomic relationships of diploids and the origins of allotetraploids in *Dactylorhiza* (Orchidaceae). *Syst. Biol.* 69 91–109. 10.1093/sysbio/syz035 31127939 PMC6902629

[B11] Bryant D. Bouckaert R. Felsenstein J. Rosenberg N. A. RoyChoudhury A. (2012). Inferring species trees directly from biallelic genetic markers: bypassing gene trees in a full coalescent A analysis. *Mol. Biol. Evol.* 29 1917–1932. 10.1093/molbev/mss086 22422763 PMC3408069

[B12] Burnham K. P. Anderson D. R. (2002). *Model Selection and Multimodel Inference. A Practical Information-Theoretic Approach.* New York, NY: Springer.

[B13] Caković D. Cresti L. Stešević D. Schönswetter P. Frajman B. (2021). High genetic and morphological diversification of the *Euphorbia verrucosa* alliance (Euphorbiaceae) in the Balkan and Iberian peninsulas. *Taxon* 70 286–307.

[B14] Caković D. Frajman B. (2020). Three tertiary *Euphorbia* species persisted in the forests of the Balkan Peninsula. *Plant Syst. Evol.* 306:50. 10.1007/s00606-020-01672-w

[B15] Carnicero P. Garcia-Jacas N. Sáez L. Constantinidis T. Galbany-Casals M. (2020). Disentangling relationships among eastern Mediterranean *Cymbalaria* including description of a novel species from the southern Peloponnese (Greece). *Plant Syst. Evol.* 307:13. 10.1007/s00606-020-01730-3

[B16] Catchen J. Hohenlohe P. Bassham S. Amores A. Cresko W. (2013). Stacks: an analysis tool set for population genomics. *Mol. Ecol.* 22 3124–3140. 10.1111/mec.12354 23701397 PMC3936987

[B17] Catchen J. M. Amores A. Hohenlohe P. Cresko W. Postlethwait J. H. (2011). Stacks: building and genotyping loci de novo from short-read sequences. *G3* 1 171–182. 10.1534/g3.111.000240 22384329 PMC3276136

[B18] Cetlová V. Zozomová-Lihová J. Melichárková A. Mártonfiová L. Španiel S. (2021). Multiple drivers of high species diversity and endemism among *Alyssum* annuals in the Mediterranean: the evolutionary significance of the Aegean hotspot. *Front. Plant Sci.* 12:627909. 10.3389/fpls.2021.627909 33986760 PMC8112278

[B19] Charles K. L. Bell R. C. Blackburn D. C. Burger M. Fujita M. K. Gvoždík V. (2018). Sky, sea, and forest islands: diversification in the African leaffolding frog *Afrixalus paradorsalis* (Anura: Hyperoliidae) of the lower Guineo- Congolian rain forest. *J. Biogeogr.* 45 1781–1794. 10.1111/jbi.13365

[B20] Clement M. Posada D. Crandall K. A. (2000). TCS: a computer program to estimate gene genealogies. *Mol. Ecol.* 9 1657–1660. 10.1046/j.1365-294x.2000.01020.x 11050560

[B21] Crowl A. A. Visger C. J. Mansion G. Hand R. Wu H. H. Kamari G. (2015). Evolution and biogeography of the endemic *Roucela* complex (Campanulaceae: *Campanula*) in the Eastern Mediterranean. *Ecol. Evol.* 5 5329–5343. 10.1002/ece3.1791 30151135 PMC6102515

[B22] Csergö A. M. Schönswetter P. Gyöngyvér M. Deák T. Boşcaiu N. Höhn M. (2009). Genetic structure of peripheral, island-like populations: a case study of *Saponaria bellidifolia* Sm. (Caryophyllaceae) from the Southeastern Carpathians. *Plant Syst. Evol.* 278 33–41. 10.1007/s00606-008-0129-5

[B23] Danecek P. Auton A. Abecasis G. Albers C. A. Banks E. DePristo M. A. (2011). The variant call format and vcftools. *Bioinformatics* 27 2156–2158. 10.1093/bioinformatics/btr330 21653522 PMC3137218

[B24] Daneck H. Fér T. Marhol K. (2016). Glacial survival in northern refugia? Phylogeography of the temperate shrub *Rosa pendulina* L. (Rosaceae): AFLP vs. chloroplast DNA variation. *Biol. J. Linn. Soc.* 119 704–718. 10.1111/bij.12619

[B25] Drummond A. J. Suchard M. A. Xie D. Rambaut A. (2012). Bayesian phylogenetics with BEAUti and the BEAST 1.7. *Mol. Biol. Evol.* 29 1969–1973. 10.1093/molbev/mss075 22367748 PMC3408070

[B26] Dudley T. R. (1964). Synopsis of the genus *Aurinia* in Turkey. *J. Arnold Arbor.* 45 390–400. 10.5962/p.325004 33311142

[B27] Đurović S. Schönswetter P. Niketić M. Tomović G. Frajman B. (2017). Disentangling relationships among the members of the *Silene saxifraga* alliance (Caryophyllaceae): phylogenetic structure is geographically rather than taxonomically segregated. *Taxon* 66 343–364. 10.12705/662.4

[B28] Elleouet J. S. Aitken S. N. (2018). Exploring approximate Bayesian computation for inferring recent demographic history with genomic markers in nonmodel species. *Mol. Ecol. Resour.* 18 525–540. 10.1111/1755-0998.12758 29356336

[B29] Falch M. Schönswetter P. Frajman B. (2019). Both vicariance and dispersal have shaped the genetic structure of Eastern Mediterranean *Euphorbia myrsinites* (Euphorbiaceae). *Perspect. Plant Ecol. Evol. Syst.* 39:125459. 10.1016/j.ppees.2019.125459

[B30] Frajman B. Graniszewska M. Schönswetter P. (2016). Evolutionary patterns and morphological diversification within the European members of the *Euphorbia illirica* (*E. villosa*) group: one or several species? *Preslia* 88 369–390.

[B31] Frajman B. Oxelman B. (2007). Reticulate phylogenetics and phytogeographical structure of *Heliosperma* (Sileneae, Caryophyllaceae) inferred from chloroplast and nuclear DNA sequences. *Mol. Phylogenet. Evol.* 43 140–155. 10.1016/j.ympev.2006.11.003 17188521

[B32] Frajman B. Schönswetter P. (2017). Amphi-Adriatic distributions in plants revisited: pleistocene trans-Adriatic dispersal in the *Euphorbia barrelieri* group (Euphorbiaceae). *Bot. J. Linn. Soc.* 185 240–252. 10.1093/botlinnean/box055

[B33] Frajman B. Záveská E. Gamisch A. Moser T., The STEPPE Consortium, and Schönswetter P. (2019). Integrating phylogenomics, phylogenetics, morphometrics, relative genome size and ecological niche modelling disentangles the diversification of Eurasian *Euphorbia seguieriana* s. l. (Euphorbiaceae). *Mol. Phylogenet. Evol.* 134 238–252. 10.1016/j.ympev.2018.10.046 30415023

[B34] Garcia-Jacas N. López-Pujol J. López-Vinyallonga S. Janaćković P. Susanna A. (2019). *Centaurea* subsect. *Phalolepis* in Southern Italy: ongoing speciation or species overestimation? Genetic evidence based on SSRs analyses. *System. Biodivers.* 17 93–109. 10.1080/14772000.2018.1549617

[B35] Gernhard T. (2008). The conditioned reconstructed process. *J. Theor. Biol.* 253 769–778. 10.1016/j.jtbi.2008.04.005 18538793

[B36] Gömöry D. Zhelev P. Brus R. (2020). The Balkans: a genetic hotspot but not a universal colonization source for trees. *Plant Syst. Evol.* 306:5. 10.1007/s00606-020-01647-x

[B37] Gutenkunst R. N. Hernandez R. D. Williamson S. H. Bustamante C. D. (2009). Inferring the joint demographic history of multiple populations from multidimensional SNP frequency data. *PLoS Genet.* 5:e1000695. 10.1371/journal.pgen.1000695 19851460 PMC2760211

[B38] Hamming R. W. (1950). Error detecting and error correcting codes. *Bell Syst. Tech. J.* 29 147–160.

[B39] Hewitt G. M. (1999). Postglacial re-colonisation of European biota. *Biol. J. Linn. Soc.* 68 87–112. 10.1186/1471-2148-11-215 21777453 PMC3155922

[B40] Hewitt G. M. (2011). “Mediterranean peninsulas: the evolution of hotspots,” in *Biodiversity Hotspots*, eds Zachos F. E. Habel J. C. (Berlin: Springer), 123–147. 10.1007/978-3-642-20992-5_7

[B41] Horvat I. Glavač V. Ellenberg H. (1974). *Vegetation Südosteuropas.* Stuttgart: Fischer.

[B42] Huang X.-C. German D. A. Koch M. A. (2020). Temporal patterns of diversification in Brassicaceae demonstrate decoupling of rate shifts and mesopolyploidization events. *Ann. Bot.* 125 29–47. 10.1093/aob/mcz123 31314080 PMC6948214

[B43] Huson D. H. Bryant D. (2006). Application of phylogenetic networks in evolutionary studies. *Mol. Biol. Evol.* 23 254–267. 10.1093/molbev/msj030 16221896

[B44] Janković I. Satovic Z. Liber Z. Kuzmanović N. Di Pietro R. Radosavljević I. (2019). Genetic and morphological data reveal new insights into the taxonomy of *Campanula versicolor* s.l. (Campanulaceae). *Taxon* 68 340–369. 10.1002/tax.12050

[B45] Jaros U. Tribsch A. Comes H. P. (2018). Diversification in continental island archipelagos: new evidence on the roles of fragmentation, colonization and gene flow on the genetic divergence of Aegean *Nigella* (Ranunculaceae). *Ann. Bot.* 121 241–254. 10.1093/aob/mcx150 29300817 PMC5808797

[B46] Jombart T. (2008). adegenet: a R package for the multivariate analysis of genetic markers. *Bioinformatics* 24 1403–1405. 10.1093/bioinformatics/btn129 18397895

[B47] Jombart T. Ahmed I. (2011). adegenet 1.3-1: new tools for the analysis of genome-wide SNP data. *Bioinformatics* 27 3070–3071. 10.1093/bioinformatics/btr521 21926124 PMC3198581

[B48] Karger D. N. Conrad O. Böhner J. Kawohl T. Kreft H. Soria-Auza R. W. (2018). Data from: climatologies at high resolution for the earth’s land surface areas. *Dryad Digit. Rep.* 10.5061/dryad.kd1d4 PMC5584396 28872642

[B49] Kirschner P. Záveská E. Gamisch A. Hilpold A. Trucchi E. Paun O. (2020). Long-term isolation of European steppe outposts boosts the biome’s conservation value. *Nat. Commun.* 11:1968. 10.1038/s41467-020-15620-2 32327640 PMC7181837

[B50] Kolář F. Fuxová G. Záveská E. Nagano A. J. Hyklová L. Lučanová M. (2016). Northern glacial refugia and altitudinal niche divergence shape genome-wide differentiation in the emerging plant model *Arabidopsis arenosa*. *Mol. Ecol.* 25 3929–3949. 10.1111/mec.13721 27288974

[B51] Kramp K. Huck S. Niketić M. Tomović G. Schmitt T. (2009). Multiple glacial refugia and complex postglacial range shifts of the obligatory woodland plant *Polygonatum verticillatum* (Convallariaceae). *Plant Biol.* 11 392–404. 10.1111/j.1438-8677.2008.00130.x 19470110

[B52] Kuzmanović N. Comanescu P. Frajman B. Lazarević M. Paun O. Schönswetter P. (2013). Genetic, cytological and morphological differentiation within the Balkan-Carpathian *Sesleria rigida* sensu Fl. Eur. (Poaceae): a taxonomically intricate tetraploid-octoploid complex. *Taxon* 62 458–472.

[B53] Kuzmanović N. Lakušić D. Frajman B. Stevanovski I. Conti F. Schönswetter P. (2021). Long neglected diversity in the Accursed Mountains (western Balkan Peninsula): *Ranunculus bertisceus* is a genetically and morphologically divergent new species. *Bot. J. Linn. Soc.* 196 384–406. 10.1093/botlinnean/boab001

[B54] Laczkó L. Sramkó G. (2020). *Hepatica transsilvanica* Fuss (Ranunculaceae) is an allotetraploid relictof the tertiary flora in Europe - Molecular phylogenetic evidence. *Acta Soc. Bot. Pol.* 89:8934. 10.5586/asbp.8934

[B55] Li H. Durbin R. (2009). Fast and accurate short read alignment with burrows-wheeler transform. *Bioinformatics* 25 1754–1760. 10.1093/bioinformatics/btp324 19451168 PMC2705234

[B56] López-Vinyallonga S. López-Pujol J. Constantinidis T. Susanna A. Garcia-Jacas N. (2015). Mountains and refuges: genetic structure and evolutionary history in closely related, endemic *Centaurea* in continental Greece. *Mol. Phylogenet. Evol.* 92 243–254. 10.1016/j.ympev.2015.06.018 26151220

[B57] Lysak M. A. Koch M. A. Beaulieu J. M. Meister A. Leitch I. J. (2009). The dynamic ups and downs of genome size evolution in Brassicaceae. *Mol. Biol. Evol.* 26 85–98. 10.1093/molbev/msn223 18842687

[B58] Magri D. Vendramin G. G. Comps B. Dupanloup I. Geburek T. Gömöry D. (2006). A new scenario for the Quaternary history of European beech populations: palaeobotanical evidence and genetic consequences. *New Phytol.* 171 199–221. 10.1111/j.1469-8137.2006.01740.x 16771995

[B59] Mansion G. Selvi F. Guggisberg A. Conti E. (2009). Origin of Mediterranean insular endemics in the Boraginales: integrative evidence from molecular dating and ancestral area reconstruction. *J. Biogeogr.* 36 1282–1296. 10.1111/j.1365-2699.2009.02082.x

[B60] McKenna A. Hanna M. Banks E. Sivachenko A. Cibulskis K. Kernytsky A. (2010). The genome analysis toolkit: a MapReduce framework for analyzing next-generation DNA sequencing data. *Genome Res.* 20 1297–1303. 10.1101/gr.107524.110 20644199 PMC2928508

[B61] Naimi B. Hamm N. A. S. Groen T. A. Skidmore A. K. Toxopeus A. G. (2014). Where is positional uncertainty a problem for species distribution modelling? *Ecography* 37 191–203. 10.1111/j.1600-0587.2013.00205.x

[B62] Nieto Feliner G. (2014). Patterns and processes in plant phylogeography in the Mediterranean Basin: a review. *Perspect. Plant Ecol. Evol. Syst.* 16 265–278. 10.1016/j.ppees.2014.07.002

[B63] Nylander J. A. A. (2004). *MrModeltest v2. Program Distributed by the Author.* Uppsala: Evolutionary Biology Centre, Uppsala University.

[B64] Pattengale N. D. Alipour M. Bininda-Emonds O. R. Moret B. M. Stamatakis A. (2010). How many bootstrap replicates are necessary? *J. Comput. Biol.* 17 337–354. 10.1089/cmb.2009.0179 20377449

[B65] Paun O. Turner B. Trucchi E. Munzinger J. Chase M. W. Samuel R. (2016). Processes driving the adaptive radiation of a tropical tree (*Diospyros*, Ebenaceae) in New Caledonia, a biodiversity hotspot. *Syst. Biol.* 65, 212–227. 10.1093/sysbio/syv076 26430059 PMC4748748

[B66] Peel M. C. Finlayson B. L. McMahon T. A. (2007). Updated world map of the Köppen-Geiger climate classification. *Hydrol. Earth Syst. Sci.* 11 1633–1644. 10.5194/hess-11-1633-2007

[B67] Persson J. (1971). Studies in the Aegean Flora XIX - Notes on *Alyssum* and some other genera of Cruciferare. *Bot. Not.* 124 399–418.

[B68] Petit R. J. Aguinagalde I. De Beaulieu J. L. Bittkau C. Brewer S. Cheddadi R. (2003). Glacial refugia: hotspots but not melting pots of genetic diversity. *Science* 300 1563–1565. 10.1126/science.1083264 12791991

[B69] Plazibat M. (2009). A short synopsis of the tribe Alysseae (Brassicaceae) in Croatia with some taxonomic novelties. *Nat. Croat.* 18 401–426.

[B70] Portik D. M. Leaché A. D. Rivera D. Barej M. F. Burger M. Hirschfeld M. (2017). Evaluating mechanisms of diversification in a Guineo-Congolian tropical forest frog using demographic model selection. *Mol. Ecol.* 26 5245–5263. 10.1111/mec.14266 28748565

[B71] Poulakakis N. Kapli P. Lymberakis P. Trichas A. Vardinoyiannis K. Sfenthourakis S. (2015). A review of phylogeographic analyses of animal taxa from the Aegean and surrounding regions. *J. Zoolog. Syst. Evol.* 53 18–32.

[B72] Puşcaş M. Choler P. Tribsch A. Gielly L. Rioux D. Gaudeul M. (2008). Post-glacial history of the dominant alpine sedge *Carex curvula* in the European Alpine system inferred from nuclear and chloroplast markers. *Mol. Ecol.* 17 2417–2429. 10.1111/j.1365-294X.2008.03751.x 18422934

[B73] Raj A. Stephens M. Pritchard J. K. (2014). fastSTRUCTURE: variational inference of population structure in large SNP data sets. *Genetics* 197 573–589. 10.1534/genetics.114.164350 24700103 PMC4063916

[B74] Rambaut A. (2014). *FigTree 1.4.2.* Available online at: http://tree.bio.ed.ac.uk/ (accessed November 10, 2020).

[B75] Rambaut A. Suchard M. A. Xie D. Drummond A. J. (2014). *Tracer v 1.6.* Available online at: http://beast.bio.ed.ac.uk/Tracer (accessed November 10, 2020).

[B76] Rešetnik I. Frajman B. Schönswetter P. (2016). Heteroploid *Knautia drymeia* includes *K. gussonei* and cannot be separated into diagnosable subspecies. *Am. J. Bot.* 103 1300–1313. 27425632 10.3732/ajb.1500506

[B77] Rešetnik I. Satovic Z. Schneeweiss G. M. Liber Z. (2013). Phylogenetic relationships in Brassicaceae tribe Alysseae inferred from nuclear ribosomal and chloroplast DNA sequence data. *Mol. Phylogenet. Evol.* 69 772–786. 10.1016/j.ympev.2013.06.026 23850498

[B78] Rieseberg L. H. Soltis D. E. (1991). Phylogenetic consequences of cytoplasmicgene flow in plants. *Evol. Trends Plants* 5 65–84.

[B79] Ronikier M. (2011). Biogeography of high-mountain plants in the Carpathians: an emerging phylogeographical perspective. *Taxon* 60 372–389.

[B80] Ronikier M. Zalewska-Gałosz J. (2014). Independent evolutionary history between the Balkan ranges and more northerly mountains in *Campanula alpina* s.l. (Campanulaceae): genetic divergence and morphological segregation of taxa. *Taxon* 63 116–131.

[B81] Ronquist F. Teslenko M. Van Der Mark P. Ayres D. L. Darling A. Höhna S. (2012). MrBayes 3.2: efficient bayesian phylogenetic inference and model choice across a large model space. *Syst. Biol.* 61 539–542. 10.1093/sysbio/sys029 22357727 PMC3329765

[B82] Slovák M. Kučera J. Turis P. Zozomová-Lihová J. (2012). Multiple glacial refugia and postglacial colonization routes inferred for a woodland geophyte, *Cyclamen purpurascens*: Patterns concordant with the Pleistocene history of broadleaved and coniferous tree species. *Biol. J. Linn. Soc.* 105 741–760.

[B83] Španiel S. Marhold K. Zozomová-Lihová J. (2017). The polyploid *Alyssum montanum-A. repens* complex in the Balkans: a hotspot of species and genetic diversity. *Plant Syst. Evol.* 303 1443–1465. 10.1007/s00606-017-1470-3

[B84] Šrámková-Fuxová G. Záveská E. Kolář F. Lučanová M. Španiel S. Marhold K. (2017). Range-wide genetic structure of *Arabidopsis halleri* (Brassicaceae): glacial persistence in multiple refugia and origin of the Northern Hemisphere disjunction. *Bot. J. Linn. Soc.* 185 321–342. 10.1093/botlinnean/box064

[B85] Stachurska-Swakoń A. Cieślak E. Ronikier M. (2013). Phylogeography of a subalpine tall-herb *Ranunculus platanifolius* (Ranunculaceae) reveals two main genetic lineages in the European mountains. *Bot. J. Linn. Soc.* 171 413–428. 10.1111/j.1095-8339.2012.01323.x

[B86] Stamatakis A. (2014). RAxML version 8: a tool for phylogenetic analysis and post-analysis of large phylogenies. *Bioinformatics* 30 1312–1313. 10.1093/bioinformatics/btu033 24451623 PMC3998144

[B87] Stange M. Sánchez-Villagra M. R. Salzburger W. Matschiner M. (2018). Bayesian divergence-time estimation with genome-wide single-nucleotide polymorphism data of sea catfishes (Ariidae) supports Miocene closure of the Panamanian Isthmus. *Syst. Biol.* 67, 681–699. 10.1093/sysbio/syy006 29385552 PMC6005153

[B88] Surina B. Schneeweiss G. M. Glasnović P. Schönswetter P. (2014). Testing the efficiency of nested barriers to dispersal in the Mediterranean high mountain plant *Edraianthus graminifolius* (Campanulaceae). *Mol. Ecol.* 23 2861–2875. 10.1111/mec.12779 24811794

[B89] Svenning J. C. (2003). Deterministic Plio-Pleistocene extinctions in the European cool-temperate tree flora. *Ecol. Lett.* 6 646–653. 10.1046/j.1461-0248.2003.00477.x

[B90] Swofford D. L. (2002). *PAUP*. Phylogenetic Analysis Using Parsimony (*and Other Methods), ver. 4.0 beta 10.* Sunderland: Sinauer Associates.

[B91] Taberlet P. Fumagalli L. Wust-Saucy A.-G. Cosson J.-F. (1998). Comparative phylogeography and postglacial colonization routes in Europe. *Mol. Ecol.* 7 453–464. 10.1046/j.1365-294x.1998.00289.x 9628000

[B92] Thuiller W. (2003). BIOMOD - optimizing predictions of species distributions and projecting potential future shifts under global change. *Glob. Chang. Biol.* 9 1353–1362. 10.1111/gcb.12728 25200636 PMC4340559

[B93] Thuiller W. Georges D. Engler R. Breiner F. (2016). *‘biomod2’: Ensemble Platform for Species Distribution Modelling.* Available online at: https://cran.r-project.org/web/packages/biomod2/biomod2.pdf (accessed June 11, 2021).

[B94] Thuiller W. Lafourcade B. Engler R. Araújo M. B. (2009). BIOMOD-a platform for ensemble forecasting of species distributions. *Ecography* 32 369–373. 10.1111/j.1600-0587.2008.05742.x

[B95] Turrill W. B. (1929). *The Plant-Life Of the Balkan Peninsula: A Phytogeographical Study.* Oxford: Clarendon.

[B96] Tzedakis P. C. Emerson B. C. Hewitt G. M. (2013). Cryptic or mystic? Glacial tree refugia in northern Europe. *Trends Ecol. Evol.* 28 696–704. 10.1016/j.tree.2013.09.001 24091207

[B97] Walas Ł Ganatsas P. Iszkuło G. Thomas P. A. Dering M. (2019). Spatial genetic structure and diversity of natural populations of *Aesculus hippocastanum* L. in Greece. *PLoS One* 14:e0226225. 10.1371/journal.pone.0226225 31826015 PMC6905551

[B98] Walden N. German D. A. Wolf E. M. Kiefer M. Rigault P. Huang X.-C. (2020). Nested whole-genome duplications coincide with diversification and high morphological disparity in Brassicaceae. *Nat. Commun.* 11:3795. 10.1038/s41467-020-1760.5-7 PMC7393125 32732942

[B99] Záveská E. Kirschner P. Frajman B. Wessely J. Willner W. Gattringer A. (2021). Evidence for glacial refugia of the forest understorey species *Helleborus niger* (Ranunculaceae) in the Southern as well as in the Northern Limestone Alps. *Front. Plant Sci.* 12:683043. 10.3389/fpls.2021.683043 34040627 PMC8141911

[B100] Záveská E. Maylandt C. Paun O. Bertel C. Frajman B. Schönswetter P. (2019). Multiple auto-and allopolyploidisations marked the Pleistocene history of the widespread Eurasian steppe plant *Astragalus onobrychis* (Fabaceae). *Mol. Phylogenet. Evol.* 139:106572. 10.1016/j.ympev.2019.106572 31351183

