# Supplementary material for: Stability in the South, Turbulence Toward the North: Evolutionary History of Aurinia saxatilis (Brassicaceae) Revealed by Phylogenomic and Climatic Modelling Data
Source: Front Plant Sci. 2022 Mar 14;13:822331. doi: 10.3389/fpls.2022.822331 (PMC8964184; doi:10.3389/fpls.2022.822331)
Supplement: Supplementary file 1 [file Presentation_1.pdf]

frontiers

Supplementary Material

Supplementary Figures

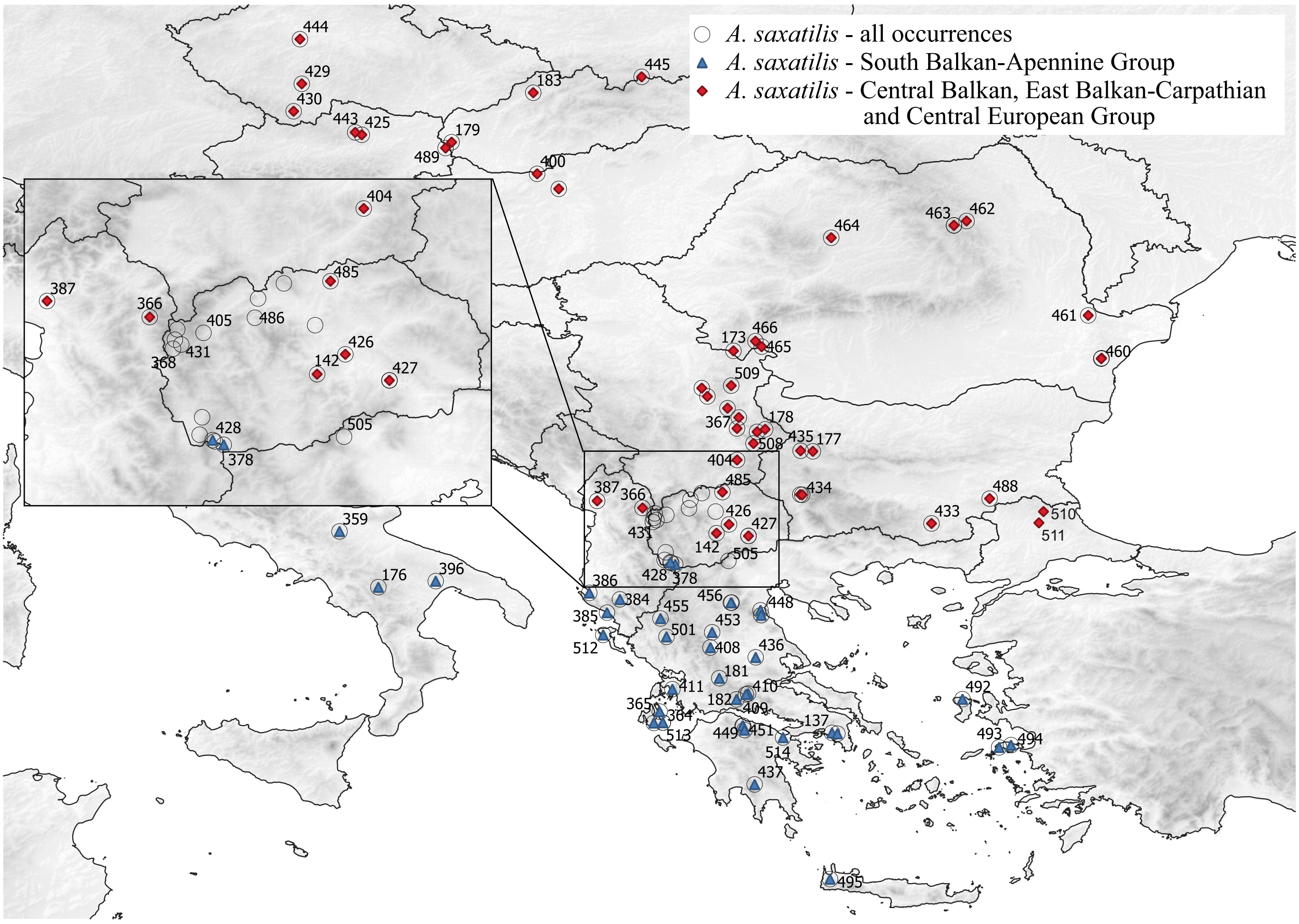

Supplementary Figure 1. Sampled populations of *Aurinia saxatilis*. Populations with identifiers were used in phylogenetic analyses and correspond to Supplementary Table 1 and were complemented with additional populations (circles without identifiers in the map) for species distribution modelling. All occurrences were used for current and Last Glacial Maximum (LGM) species distribution modelling (SDM) of the entire dataset, while color coding was used for current and LGM SDMs of separate analyses of red – Central Balkan, East Balkan-Carpathian and Central European Group and blue – South Balkan-Apennine Group.

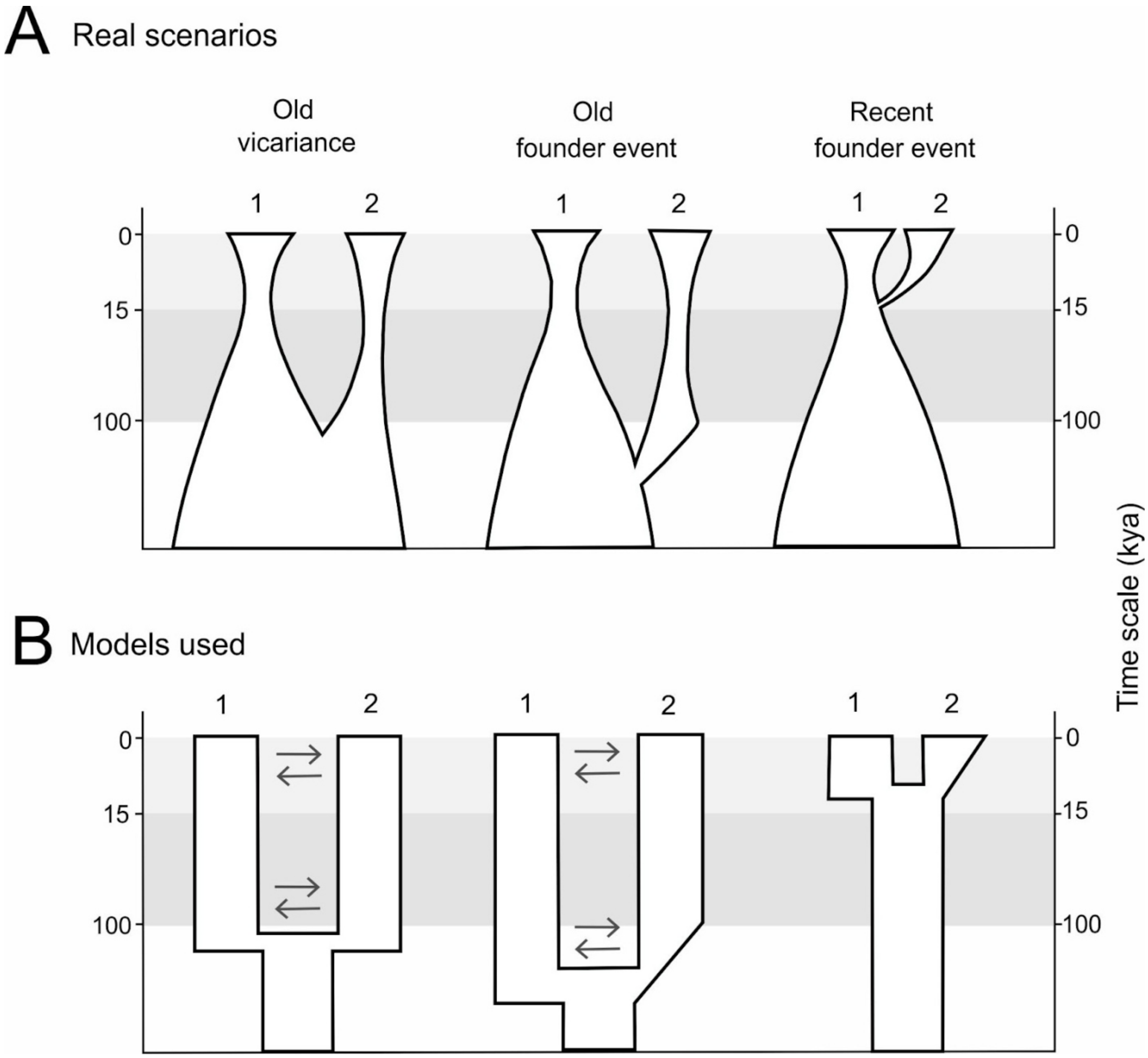

Supplementary Figure 2. (**A**) Scenarios explaining adjacent distribution of *Aurinia saxatilis*. Left and middle pane present a pre-glacial vicariance or founder event leading to split of ancestral population into current populations 1 and 2. Right pane presents a post-glacial founder event giving rise to population 2 from (ancestral) population 1. (**B**) Demographic models summarising particular scenarios in (A), tested using dadi. All models tested are shown and described in Supplementary Figure 3. Numbers 1 and 2 refer to current populations (i.e. genetic groups defined based on fastSTRUCTURE analysis); gene flow is indicated by arrows.

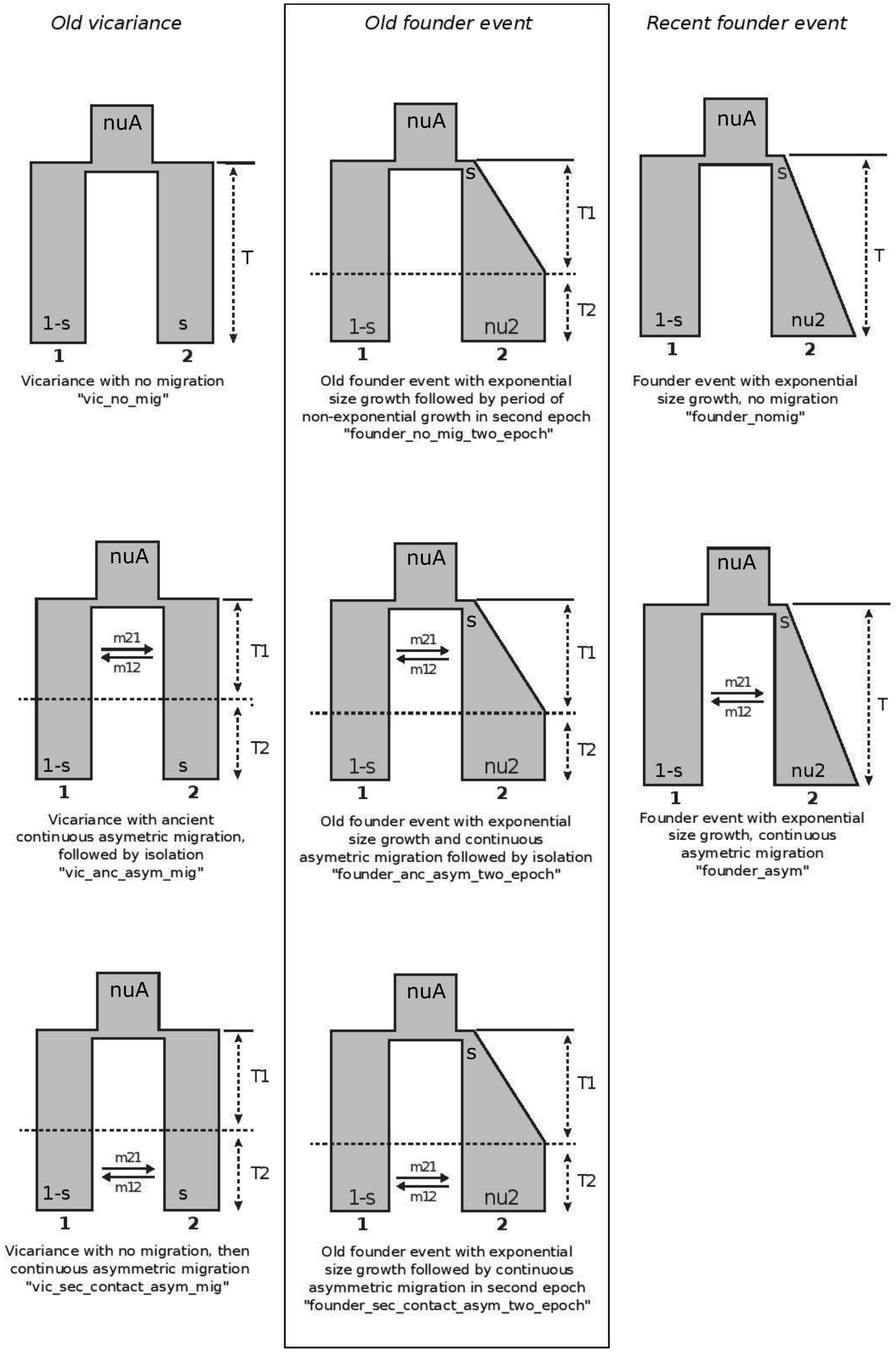

Supplementary Figure 3. Models used for 2D demographic modeling with dadi. Framed models were taken from Záveská et al. (2021), while unframed models were taken from Charles et al. (2018). Numbers 1 and 2 refer to current populations; gene flow (m – migration) is indicated by arrows. Variable "s" defines the fraction of the ancestral population (nuA) founding each daughter population, where nuA*s represents the founded population and nuA*(1-s) represents the founding ancestral population.

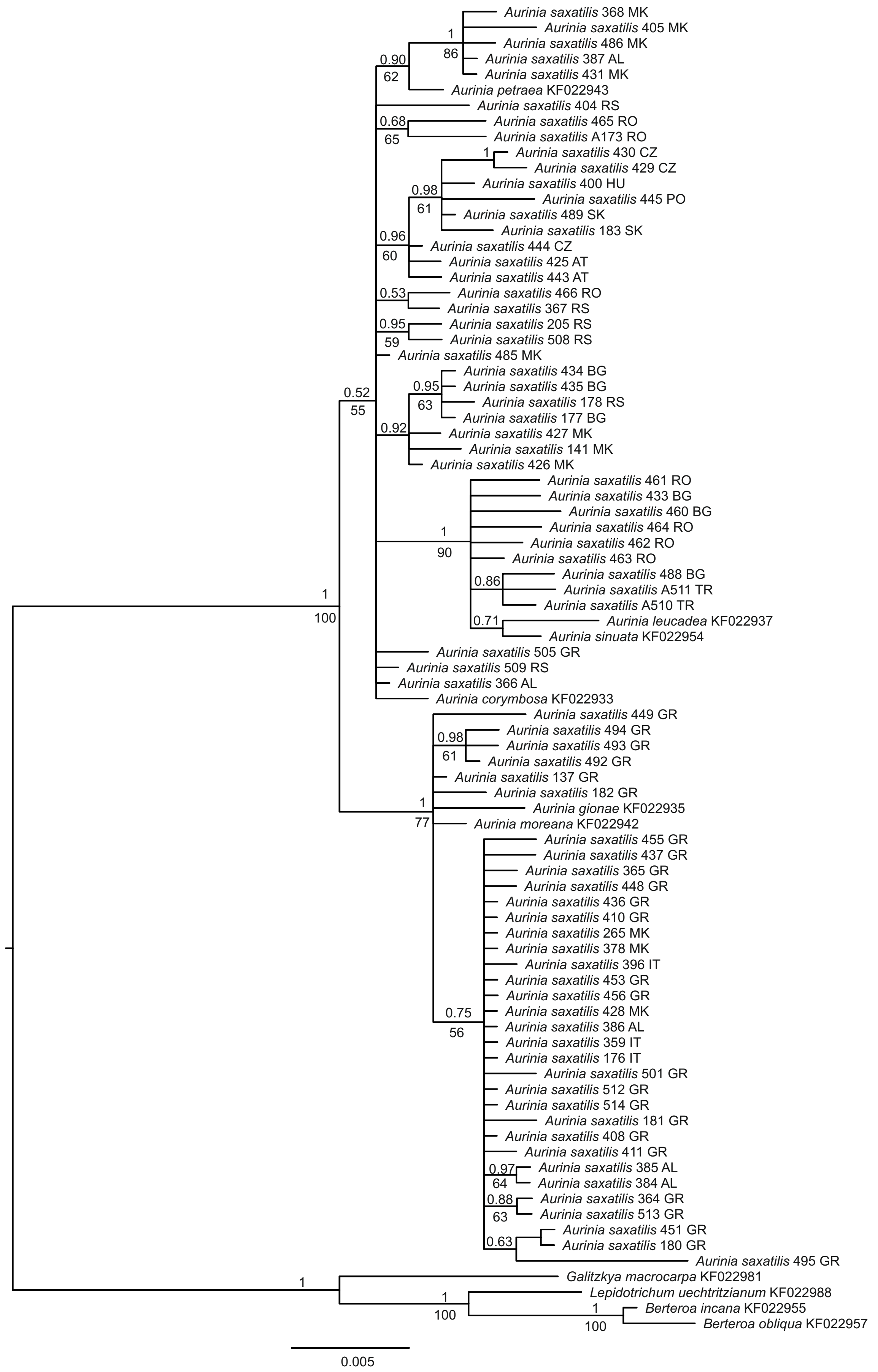

Supplementary Figure 4. Bayesian consensus phylogram inferred from phylogenetic analyses of plastid *ndh*F sequences of *Aurinia saxatilis* and closely related species. Numbers above branches are PP values > 0.50 and those below branches bootstrap support values >50 based on Maximum parsimony analysis. Population identifiers of *A. saxatilis* correspond to Supplementary Table 1 and Supplementary Figure 1 and are followed by ISO country codes; in case of other *Aurinia* as well as outgroup species the taxon names are supplemented by Genbank numbers.

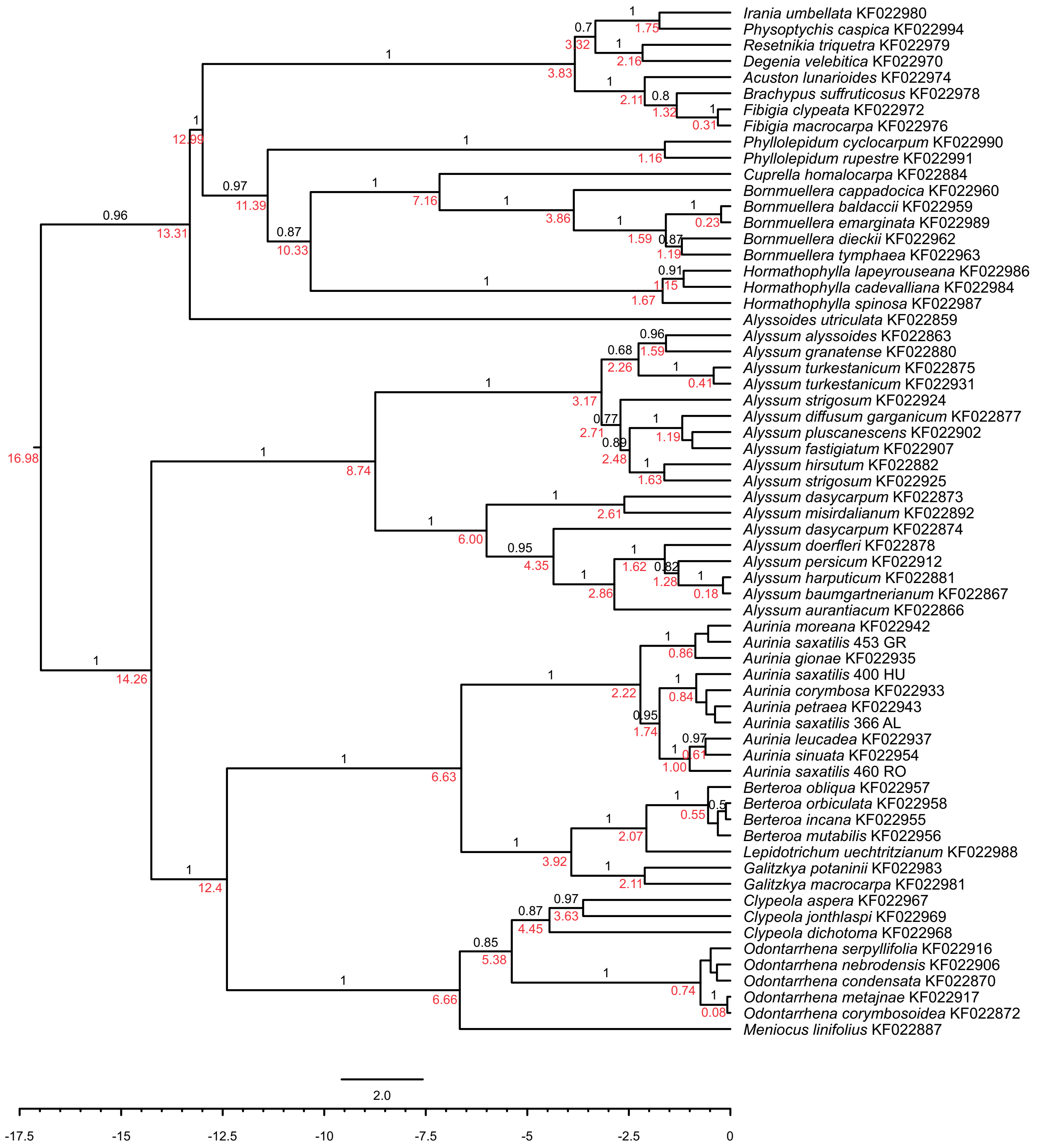

Supplementary Figure 5. Maximum clade credibility tree generated from the relaxed molecular-clock analysis in BEAST based on *ndh*F sequence data of the tribe Alysseae. Time scale is in millions of years, values above branches are Bayesian posterior probability values > 0.80 and values below branches (in red) are medians of divergence time estimates. Accession names are supplemented with Genbank numbers and in the case of *Aurinia saxatilis* with identifiers as in Supplementary Table 1 and Supplementary Figure 1.

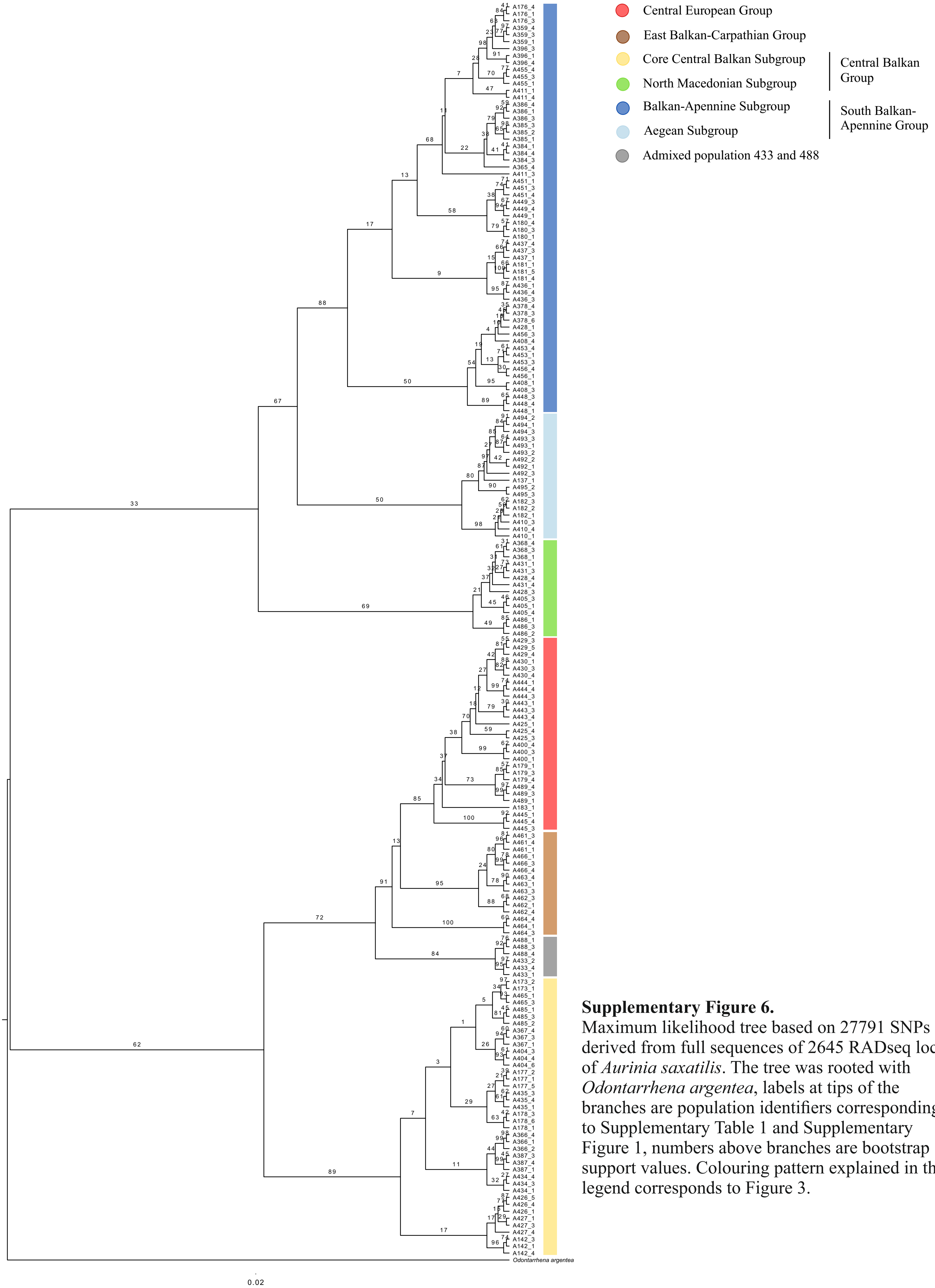

Supplementary Figure 6.
Maximum likelihood tree based on 27791 SNPs derived from full sequences of 2645 RADseq loci of *Aurinia saxatilis*. The tree was rooted with *Odontarrhena argentea*, labels at tips of the branches are population identifiers corresponding to Supplementary Table 1 and Supplementary Figure 1, numbers above branches are bootstrap support values. Colouring pattern explained in the legend corresponds to Figure 3.

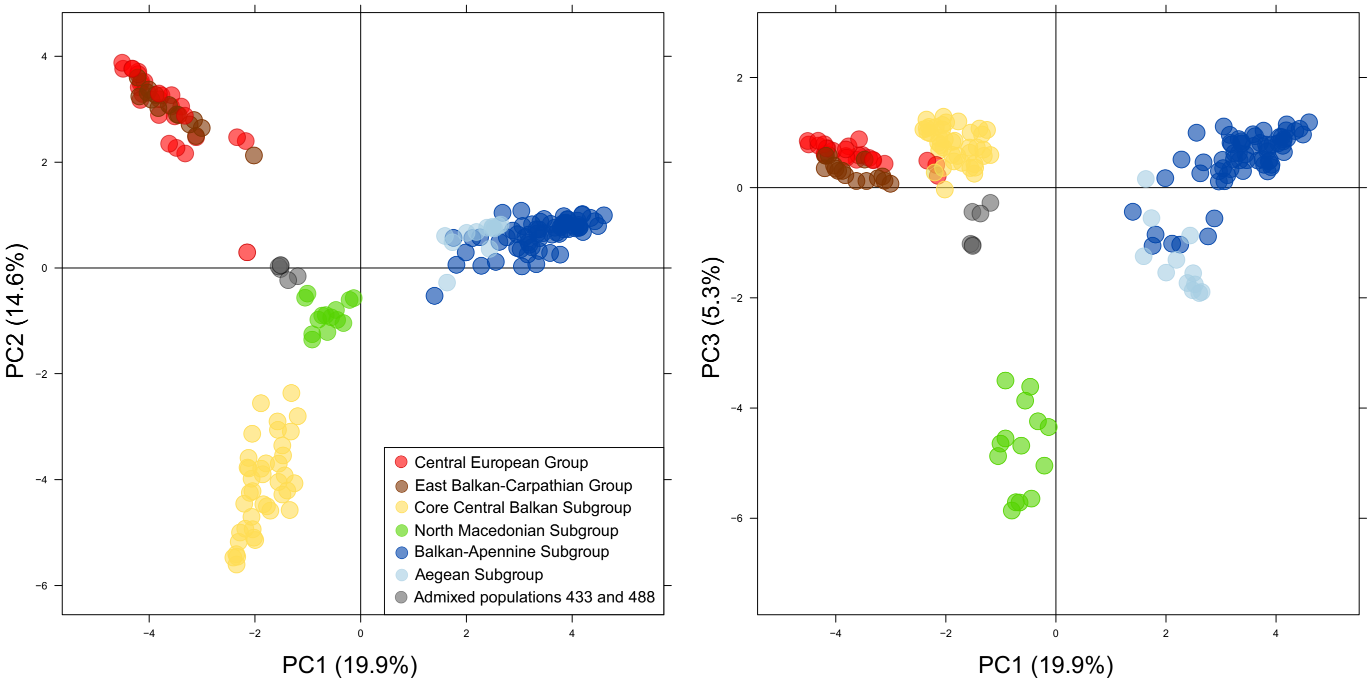

Supplementary Figure 7. Principal coordinate analysis of 2279 unlinked SNPs derived from RADseq loci of *Aurinia saxatilis*. The variation along the first and the second (left) and the third (right) principal coordinates is displayed, and the percentage of explained variance along them indicated. Colouring pattern explained in the legend corresponds to Figure 3 and Supplementary Figure 5.

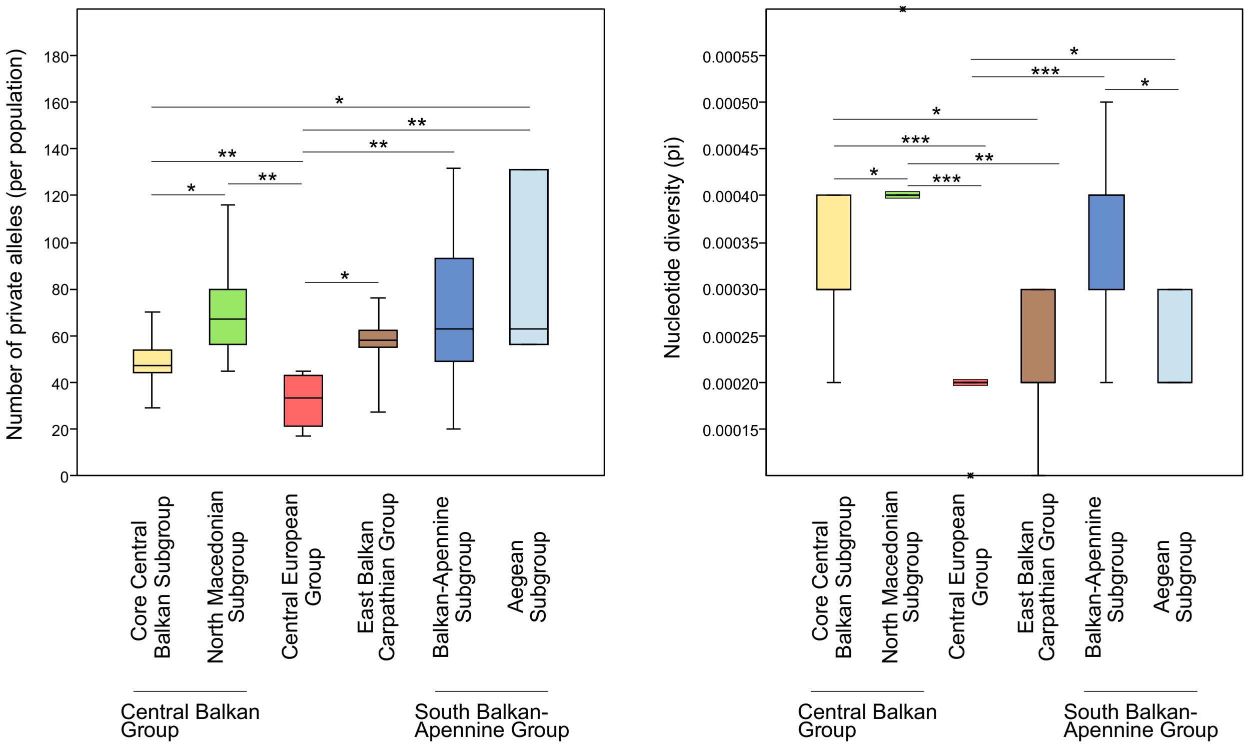

Supplementary Figure 8. Comparison of the number of private alleles (left pane) and the nucleotide diversity (π) per population (right pane) across the phylogeographic groups identified in *Aurinia saxatilis* based on RADseq data (see Figure 3). Results of Mann-Whitney significance tests of pairwise differences in distributions between groups are indicated by asterisks (***, p-valuey < 0.001; **, p < 0.01; *, p < 0.05).

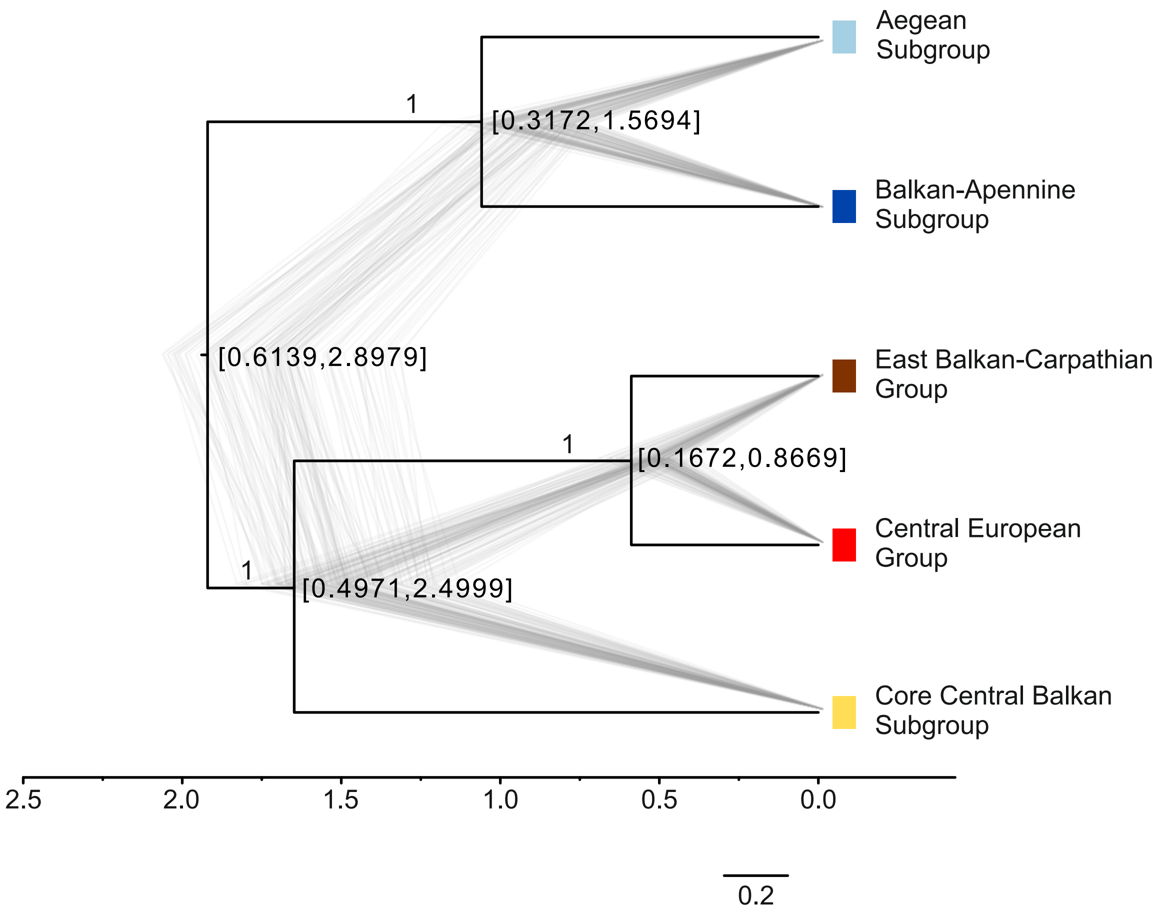

Supplementary Figure 9. Time-calibrated maximum clade credibility species tree inferred using SNAPP, visualising alternative topologies displayed with DensiTree. Time scale is given in millions of years, values above branches are Bayesian posterior probabilities and those associated to nodes and indicated in square parentheses represent 95% highest posterior densities of the node heights (ages),

frontiers

Supplementary Tables

Supplementary Table 1. Locality and collection details of sampled populations of *Aurinia saxatilis*. Number of individuals per population used in RADseq analyses, populations used in species distribution modelling (SDM), *ndh*F Genbank and RADseq SRA accession numbers are listed. Reg – ISO country codes, ID – population identifier numbers.

Reg	ID	Genus	sp	Country	Locality	Collector	Date of collection	Voucher	Coordinate x	Coordinate y	ndhF GenBank no.	RAD seq	RADseq SRA accession no.	SDM
GR	137	*Aurinia*	*saxatilis*	Greece	Athens	Bogdanović	20.08.2007	ZA-59694	37° 58' 14.448"	23° 43' 21.972"	KF022948	1	SRR15735951	yes
MK	141	*Aurinia*	*saxatilis*	North Macedonia	Demir Kapijska klisura	Alegro	19.04.2008	no voucher	41° 24' 22.824"	22° 15' 55.584"	KF022946	no		yes
MK	142	*Aurinia*	*saxatilis*	North Macedonia	Taorska klisura	Alegro	19.04.2008	no voucher	41° 27' 14.004"	21° 42' 24.012"	no	3	SRR15735947<br>SRR15735948<br>SRR15735949	yes
RO	173	*Aurinia*	*saxatilis*	Romania	Muntii Almajului, Bigar	Španiel, Marhold, Lihová, Kolarčik	04.05.2007	ZA-58558	44° 38' 7.08"	22° 0' 8.748"	OK181034	2	SRR15735944<br>SRR15735945	yes
IT	176	*Aurinia*	*saxatilis*	Italy	Abriola, Potenza	Španiel, Perny, Kolarčik	07.06.2007	ZA-58561	40° 30' 44.892"	15° 48' 16.308"	OK181044	3	SRR15736012<br>SRR15736103<br>SRR15736104	yes
BG	177	*Aurinia*	*saxatilis*	Bulgaria	Rebrovo	Španiel, Marhold, Lihová, Kolarčik	08.05.2007	ZA-58559	42° 52' 55.128"	23° 23' 17.88"	OK181036	3	SRR15735941<br>SRR15735942<br>SRR15735943	yes
RS	178	*Aurinia*	*saxatilis*	Serbia	Topli do, Pirot	Španiel, Šibik	17.05.2008	ZA-56563	43° 15' 58.212"	22° 33' 19.404"	OK181023	3	SRR15735938<br>SRR15735939<br>SRR15735940	yes
SK	179	*Aurinia*	*saxatilis*	Slovakia	castle Pajštun, Stupava	Španiel, Mereďa, Šingliarová	22.05.2008	ZA-58557	48° 16' 32.988"	17° 4' 59.016"	no	3	SRR15736057<br>SRR15736059<br>SRR15736060	yes
GR	180	*Aurinia*	*saxatilis*	Greece	Kato Zachlorou village	Španiel, Marhold, Lihová	29.05.2008	ZA-58555	38° 5' 28.608"	22° 10' 16.788"	KF022949	3	SRR15735979<br>SRR15735990<br>SRR15736001	yes
GR	181	*Aurinia*	*saxatilis*	Greece	Karpanisi	Španiel, Marhold, Lihová	02.06.2008	ZA-58556	38° 55' 29.568"	21° 45' 18.108"	OK181048	3	SRR15735946<br>SRR15735957<br>SRR15735968	yes
GR	182	*Aurinia*	*saxatilis*	Greece	Krokillo	Španiel, Marhold, Lihová	01.06.2008	ZA-58564	38° 33' 19.152"	22° 3' 22.68"	OK181043	3	SRR15735935<br>SRR15735950<br>SRR15736102	yes
SK	183	*Aurinia*	*saxatilis*	Slovakia	Zaskalie, Maninska tiesnava	Španiel, Lihova	27.05.2006	ZA-58565	49° 8' 22.668"	18° 30' 29.052"	OK181056	1	SRR15736061	yes
RS	205	*Aurinia*	*saxatilis*	Serbia	Sinjac, Božin grob	Niketić	04.04.2009	ZA-59648	43° 13' 18.588"	22° 24' 51.264"	KF022947	no		yes
MK	265	*Aurinia*	*saxatilis*	North Macedonia	Stenje, Prespansko jezero	Rešetnik	11.07.2009	ZA-58421	40° 56' 8.772"	20° 55' 3.288"	OK180992	no		yes

Reg	ID	Genus	sp	Country	Locality	Collector	Date of collection	Voucher	Coordinate x	Coordinate y	ndhF GenBank no.	RAD seq	RADseq SRA accession no.	SDM
IT	359	*Aurinia*	*saxatilis*	Italy	Volturino	Bogdanović, Frajman	25.06.2011	ZA-58590	41° 29' 7.584"	15° 7' 34.176"	OK181003	3	SRR15736069<br>SRR15736080<br>SRR15736091	yes
GR	364	*Aurinia*	*saxatilis*	Greece	Cephalonia, Póros	Gutermann, Berger, Flatscher, Gilli, Hofbauer	28.04.2012	WU 0091739	38° 8' 58.884"	20° 46' 12.396"	OK180995	no		yes
GR	365	*Aurinia*	*saxatilis*	Greece	Ithaka, Mon. Taxiarchón	Gutermann, Berger, Flatscher, Gilli, Hofbauer	30.04.2012	WU 0102354	38° 20' 27.888"	20° 42' 53.784"	OK180988	1	SRR15736058	yes
AL	366	*Aurinia*	*saxatilis*	Albania	Rrethi i Kukësit, Lapa	Bogdanović, Jug-Dujaković	13.07.2012	ZA-58408	41° 53' 39.192"	20° 24' 54.684"	OK181041	3	SRR15735934<br>SRR15735936<br>SRR15735937	yes
RS	367	*Aurinia*	*saxatilis*	Serbia	Soko Banja, Soko Grad	Vukojičić, Kuzmanović, Jušković	02.06.2011	ZA-60994	43° 38' 4.704"	21° 53' 55.716"	OK181038	3	SRR15735931<br>SRR15735932<br>SRR15735933	yes
MK	368	*Aurinia*	*saxatilis*	North Macedonia	road Dzhepishte - Lukovo	Bogdanović, Jug-Dujaković	13.07.2012	ZA-58402	41° 39' 0.9"	20° 35' 42.396"	OK181008	3	SRR15736068<br>SRR15736070<br>SRR15736071	yes
MK	378	*Aurinia*	*saxatilis*	North Macedonia	Konjsko	Bogdanović, Jug-Dujaković	24.08.2012	ZA-58396	40° 54' 34.92"	20° 59' 10.212"	OK180997	3	SRR15736025<br>SRR15736036<br>SRR15736047	yes
AL	384	*Aurinia*	*saxatilis*	Albania	Rrethi i Tepelenës, castle of Tepelenë	Rešetnik, Bogdanović, Temunović	24.06.2013	ZA-58371	40° 17' 59.496"	20° 1' 13.476"	OK180996	3	SRR15736011<br>SRR15736013<br>SRR15736014	yes
AL	385	*Aurinia*	*saxatilis*	Albania	Rrethi i Vlorës, Qeparo	Rešetnik, Bogdanović, Temunović	25.06.2013	ZA-58411	40° 4' 4.26"	19° 47' 43.116"	OK180994	3	SRR15736008<br>SRR15736009<br>SRR15736010	yes
AL	386	*Aurinia*	*saxatilis*	Albania	Rrethi i Vlorës, Vlorë	Rešetnik, Bogdanović, Temunović	25.06.2013	ZA-58409	40° 24' 44.064"	19° 29' 7.728"	OK181002	3	SRR15736005<br>SRR15736006<br>SRR15736007	yes
AL	387	*Aurinia*	*saxatilis*	Albania	Rrethi i Shkodrës, Mjedë	Rešetnik, Bogdanović, Temunović	26.06.2013	ZA-58412	42° 1' 6.24"	19° 37' 24.168"	OK181018	3	SRR15735928<br>SRR15735929<br>SRR15735930	yes
IT	396	*Aurinia*	*saxatilis*	Italy	Taranto, Gravina di Laterza	DiPietro	s.n.	no voucher	40° 37' 4.944"	16° 48' 20.484"	OK180998	3	SRR15736002<br>SRR15736003<br>SRR15736004	yes
HU	400	*Aurinia*	*saxatilis*	Hungary	Mt Öreg-kő, Bajót		30.05.2014	BP coll. Nr. 23552	47° 43' 26.4"	18° 34' 32.268"	OK181014	3	SRR15736054<br>SRR15736055<br>SRR15736056	yes
RS	404	*Aurinia*	*saxatilis*	Serbia	road Niš-Vranje	Rešetnik, Bogdanović, Jug-Dujaković, Ježić	19.06.2014	ZA-56345	42° 43' 53.472"	22° 3' 58.86"	OK181009	3	SRR15735925<br>SRR15735926<br>SRR15735927	yes
MK	405	*Aurinia*	*saxatilis*	North Macedonia	Mt Šar planina, Vrutok	Rešetnik, Bogdanović, Jug-Dujaković, Ježić	20.06.2014	ZA-58365	41° 46' 22.8"	20° 49' 49.404"	OK181011	3	SRR15736099<br>SRR15736100<br>SRR15736101	yes

Reg	ID	Genus	sp	Country	Locality	Collector	Date of collection	Voucher	Coordinate x	Coordinate y	ndhF GenBank no.	RAD seq	RADseq SRA accession no.	SDM
GR	408	*Aurinia*	*saxatilis*	Greece	Pili	Rešetnik, Bogdanović, Jug-Dujaković, Ježić	24.06.2014	ZA-58350	39° 27' 35.568"	21° 36' 3.024"	OK181049	3	SRR15735998 SRR15735999 SRR15736000	yes
GR	410	*Aurinia*	*saxatilis*	Greece	Sikiá	Rešetnik, Bogdanović, Jug-Dujaković, Ježić	25.06.2014	ZA-58343	38° 39' 21.528"	22° 15' 31.176"	OK180991	3	SRR15735995 SRR15735996 SRR15735997	yes
GR	411	*Aurinia*	*saxatilis*	Greece	Acarnania, Kandila	Rešetnik, Bogdanović	26.06.2014	ZA-58357	38° 43' 56.1"	20° 56' 11.688"	OK181050	3	SRR15735992 SRR15735993 SRR15735994	yes
AT	425	*Aurinia*	*saxatilis*	Austria	Wachau, Dürnstein	Pachschwöll, Laciny	03.06.2014	WU 0087197	48° 24' 20.484"	15° 30' 55.908"	OK181015	3	SRR15736051 SRR15736052 SRR15736053	yes
MK	426	*Aurinia*	*saxatilis*	North Macedonia	Lozovo, Bregalnica	Tomović, Đurović, Buzurović	06.07.2014	ZA-59545	41° 36' 25.164"	21° 55' 28.308"	OK181024	3	SRR15736096 SRR15736097 SRR15736098	yes
MK	427	*Aurinia*	*saxatilis*	North Macedonia	Valandovo, Demir Kapija	Tomović, Đurović, Buzurović	06.07.2014	ZA-59540	41° 24' 22.14"	22° 15' 50.436"	OK181022	3	SRR15736093 SRR15736094 SRR15736095	yes
MK	428	*Aurinia*	*saxatilis*	North Macedonia	Stenje, Prespansko jezero	Kolář	11.06.2014	ZA-59325	40° 56' 44.268"	20° 54' 4.788"	OK181001	3	SRR15735952 SRR15736090 SRR15736092	yes
CZ	429	*Aurinia*	*saxatilis*	Czech Republic	Bechyně	Kolář	10.08.2015	ZA-59385	49° 17' 42.864"	14° 28' 5.16"	OK181013	3	SRR15736048 SRR15736049 SRR15736050	yes
CZ	430	*Aurinia*	*saxatilis*	Czech Republic	Český Krumlov	Kolář	22.06.2014	ZA-59324	48° 48' 57.096"	14° 19' 31.944"	OK181012	3	SRR15736044 SRR15736045 SRR15736046	yes
MK	431	*Aurinia*	*saxatilis*	North Macedonia	Bistra, Sence	Tomović, Đurović	12.07.2014	ZA-59533	41° 40' 59.16"	20° 39' 30.96"	OK181037	3	SRR15736072 SRR15736073 SRR15736074	yes
BG	433	*Aurinia*	*saxatilis*	Bulgaria	Kardzhali, Monyak	Niketić, Tomović, Đurović, Buzurović	26.07.2014	ZA-59537	41° 37' 26.4"	25° 27' 34.344"	OK181026	3	SRR15736065 SRR15736066 SRR15736067	yes
BG	434	*Aurinia*	*saxatilis*	Bulgaria	Mt Rila, village Pastra	Niketić, Tomović, Đurović, Buzurović	29.07.2014	ZA-59543	42° 7' 23.808"	23° 12' 5.796"	OK181020	3	SRR15736087 SRR15736088 SRR15736089	yes
BG	435	*Aurinia*	*saxatilis*	Bulgaria	Sofia, Beledie Han	Niketić, Tomović, Đurović, Buzurović	31.07.2014	ZA-59538	42° 53' 34.44"	23° 10' 26.796"	OK181021	3	SRR15736084 SRR15736085 SRR15736086	yes
GR	436	*Aurinia*	*saxatilis*	Greece	Larisa, Farsala	Niketić, Tomović, Đurović, Buzurović	07.08.2014	ZA-59548	39° 17' 17.088"	22° 23' 24.108"	OK180990	3	SRR15735988 SRR15735989 SRR15735991	yes
GR	437	*Aurinia*	*saxatilis*	Greece	Laconia, Sparti, Mystras	Niketić, Tomović, Đurović, Buzurović	10.08.2014	ZA-59536	37° 4' 13.944"	22° 22' 19.56"	OK180987	3	SRR15735985 SRR15735986 SRR15735987	yes

Reg	ID	Genus	sp	Country	Locality	Collector	Date of collection	Voucher	Coordinate x	Coordinate y	ndhF GenBank no.	RAD seq	RADseq SRA accession no.	SDM
AT	443	*Aurinia*	*saxatilis*	Austria	Waldviertel, Purkersdorf	Pachschwöll	28.08.2014	WU 0087193	48° 26' 50.496"	15° 24' 0.216"	OK181035	3	SRR15736041 SRR15736042 SRR15736043	yes
CZ	444	*Aurinia*	*saxatilis*	Czech Republic	Prague, Vyšehrad castle	Rešetnik	06.04.2015	ZA-59526	50° 4' 25.788"	14° 26' 9.852"	OK181051	3	SRR15736038 SRR15736039 SRR15736040	yes
PL	445	*Aurinia*	*saxatilis*	Poland	Pieniny Mts, Czorsztyn castle	Wrobel	30.07.2014	no voucher	49° 25' 0.012"	20° 23' 60"	OK181052	3	SRR15736034 SRR15736035 SRR15736037	yes
GR	448	*Aurinia*	*saxatilis*	Greece	Olympus Mt., road Prionia - Lithohosio	Rešetnik, Bogdanović, Buzurević, Ljubičić	21.06.2015	ZA-60750	40° 6' 32.112"	22° 28' 29.388"	OK180989	3	SRR15735982 SRR15735983 SRR15735984	yes
GR	449	*Aurinia*	*saxatilis*	Greece	Vouraikos gorge, village Zachlorou	Rešetnik, Bogdanović, Buzurević, Ljubičić	22.06.2015	ZA-60757	38° 5' 42.9"	22° 9' 44.1"	OK181004	3	SRR15735978 SRR15735980 SRR15735981	yes
GR	451	*Aurinia*	*saxatilis*	Greece	Chelmos, Mesorrougi	Rešetnik, Bogdanović, Buzurević, Ljubičić	23.06.2015	ZA-60657	38° 1' 12.216"	22° 11' 45.204"	OK180993	3	SRR15735975 SRR15735976 SRR15735977	yes
GR	453	*Aurinia*	*saxatilis*	Greece	Meteora	Rešetnik, Bogdanović, Buzurević, Ljubičić	27.06.2015	ZA-60749	39° 43' 30.108"	21° 37' 49.584"	OK180999	3	SRR15735972 SRR15735973 SRR15735974	yes
GR	455	*Aurinia*	*saxatilis*	Greece	Mt Timfi, village Micro Papingo	Rešetnik, Bogdanović, Buzurević	28.06.2015	ZA-60658	39° 58' 0.3"	20° 43' 44.688"	OK180986	3	SRR15735969 SRR15735970 SRR15735971	yes
GR	456	*Aurinia*	*saxatilis*	Greece	Kozani, bridge Nearida - Servia	Rešetnik, Bogdanović, Buzurević, Ljubičić	28.06.2015	ZA-60659	40° 14' 31.632"	21° 57' 26.676"	OK181000	3	SRR15735965 SRR15735966 SRR15735967	yes
RO	460	*Aurinia*	*saxatilis*	Romania	Podişul Dobrogei, manastir Goru	Rešetnik, Bogdanović, Buzurević, Đurović	26.06.2016	ZA-60758	44° 29' 57.012"	28° 25' 17.004"	OK181027	no		yes
RO	461	*Aurinia*	*saxatilis*	Romania	Macin, Culmea Pricopanului	Rešetnik, Bogdanović	26.06.2016	ZA-60787	45° 15' 15.048"	28° 11' 39.948"	OK181025	3	SRR15736031 SRR15736032 SRR15736033	yes
RO	462	*Aurinia*	*saxatilis*	Romania	Neamt, Bicaz	Rešetnik, Bogdanović, Buzurević, Đurović	27.06.2016	ZA-60785	46° 54' 4.32"	26° 4' 14.592"	OK181029	3	SRR15736028 SRR15736029 SRR15736030	yes
RO	463	*Aurinia*	*saxatilis*	Romania	Neamt, Bicaz-Chei	Rešetnik, Bogdanović, Buzurević, Đurović	27.06.2016	ZA-60776	46° 49' 27.84"	25° 51' 4.5"	OK181030	3	SRR15736024 SRR15736026 SRR15736027	yes
RO	464	*Aurinia*	*saxatilis*	Romania	Turda, Cheia Tutului	Rešetnik, Bogdanović	28.06.2016	ZA-60781	46° 36' 33.516"	23° 42' 32.4"	OK181028	3	SRR15736021 SRR15736022 SRR15736023	yes
RO	465	*Aurinia*	*saxatilis*	Romania	Orsova, Virciorova	Rešetnik, Bogdanović, Buzurević, Đurović	30.06.2016	ZA-60765	44° 42' 32.832"	22° 29' 40.2"	OK181010	3	SRR15736082 SRR15736083	yes

Reg	ID	Genus	sp	Country	Locality	Collector	Date of collection	Voucher	Coordinate x	Coordinate y	ndhF GenBank no.	RAD seq	RADseq SRA accession no.	SDM
RO	466	*Aurinia*	*saxatilis*	Romania	Toplet	Rešetnik, Bogdanović, Buzurević, Đurović	30.06.2016	ZA-60783	44° 48' 16.704"	22° 23' 11.796"	OK181017	3	SRR15736018<br>SRR15736019<br>SRR15736020	yes
MK	485	*Aurinia*	*saxatilis*	North Macedonia	Novo Negovičani	Alegro	21.04.2008	ZA-59328	42° 10' 17.256"	21° 48' 35.604"	OK181019	3	SRR15736078<br>SRR15736079<br>SRR15736081	yes
MK	486	*Aurinia*	*saxatilis*	North Macedonia	Treska canyon	Alegro	20.04.2008	no voucher	41° 53' 20.832"	21° 13' 35.22"	OK181053	3	SRR15736075<br>SRR15736076<br>SRR15736077	yes
BG	488	*Aurinia*	*saxatilis*	Bulgaria	Topolovgrad, valley of Tundja river	Španiel, Mereďa	11.04.2015	SAV-448TLG-sax	42° 3' 28.08"	26° 28' 25.068"	OK181054	3	SRR15736062<br>SRR15736063<br>SRR15736064	yes
SK	489	*Aurinia*	*saxatilis*	Slovakia	Bratislava, Devin castle	Rešetnik	30.09. 2017	ZA-60995	48° 10' 27.552"	16° 58' 40.476"	OK181016	3	SRR15736015<br>SRR15736016<br>SRR15736017	yes
GR	492	*Aurinia*	*saxatilis*	Greece	Chios, Mt Pelineo, Spartounta	Španiel	21.04.2018	SAV-594PEO-sax	38° 33' 30.348"	25° 59' 58.74"	OK181007	3	SRR15735962<br>SRR15735963<br>SRR15735964	yes
GR	493	*Aurinia*	*saxatilis*	Greece	Samos, Oros Kerkis, Evangelistrias	Španiel	23.04.2018	SAV-595KEK-sax	37° 43' 3.396"	26° 38' 17.736"	OK181006	3	SRR15735959<br>SRR15735960<br>SRR15735961	yes
GR	494	*Aurinia*	*saxatilis*	Greece	Samos, Oros Ambelos, Vourliotes	Španiel	24.04.2018	SAV-596AML-sax	37° 45' 24.696"	26° 50' 50.28"	OK181005	3	SRR15735955<br>SRR15735956<br>SRR15735958	yes
GR	495	*Aurinia*	*saxatilis*	Greece	Crete, Topolia gorge	Cambria	28.02.2018	ZAGR-49870	35° 25' 7.752"	23° 41' 15.504"	OK181042	3	SRR15735953<br>SRR15735954	yes
GR	501	*Aurinia*	*saxatilis*	Greece	Ioaninna	Bogdanović, Ljubičić, Rešetnik, Terlević	22.06.2019	ZA-60648	39° 38' 49.272"	20° 49' 59.7"	OK181046	no		yes
GR	505	*Aurinia*	*saxatilis*	Greece	Loutra Loutrakiou	Bogdanović, Grgurev, Rešetnik	15.07.2019	ZA-60641	40° 58' 18.696"	21° 54' 51.912"	OK181039	no		yes
RS	508	*Aurinia*	*saxatilis*	Serbia	Grncar, Luznica river	Bogdanović, Grgurev, Rešetnik	18.07.2019	ZA-59319	43° 1' 11.712"	22° 21' 5.724"	OK181031	no		yes
RS	509	*Aurinia*	*saxatilis*	Serbia	Lazarev kanjon, Lazareva pećina	Bogdanović, Grgurev, Rešetnik	19.07.2019	ZA-59315	44° 1' 44.4"	21° 57' 48.708"	OK181040	no		yes
TR	510	*Aurinia*	*saxatilis*	Turkey	Kırklareli, Demirkapı hill	Dönmez, Mutlu	20.06.1999	AAD-7039			OK181033	no		no
TR	511	*Aurinia*	*saxatilis*	Turkey	Kırklareli, from Kırklareli to Kofçaz	Dönmez	21.04.2019	AAD-20326			OK181032	no		no
GR	512	*Aurinia*	*saxatilis*	Greece	Island Kerkyra, Gefira	Bogdanović, Brullo, Cambria	24.09.2019	ZA-62594	39° 40' 45.84"	19° 43' 46.704"	OK181055	no		yes
GR	513	*Aurinia*	*saxatilis*	Greece	Island Kefalonia, Poros	Bogdanović, Brullo, Cambria	26.09.2019	ZA-62595	38° 8' 57.588"	20° 46' 11.136"	OK181045	no		yes
GR	514	*Aurinia*	*saxatilis*	Greece	Korinth, Akrocorinth (Old Korinth)	Bogdanović, Brullo, Cambria	29.09.2019	ZA-62593	37° 53' 22.632"	22° 52' 6.888"	OK181047	no		yes

Reg	ID	Genus	sp	Country	Locality	Collector	Date of collection	Voucher	Coordinate x	Coordinate y	ndhF GenBank no.	RAD seq	RADseq SRA accession no.	SDM
		Aurinia	*saxatilis*	Hungary	Budaors, Tarokugrato	Somlyay	28.04.2001	BP-629655	47° 27' 43.704"	18° 57' 10.656"	no	no		yes
		Aurinia	*saxatilis*	Serbia	Parpćin, Mt Kučaj	Niketić, Tomović	21.04.2005	ZA-59649	43° 50' 25.404"	21° 32' 49.056"	no	no		yes
		Aurinia	*saxatilis*	Bulgaria	Rila village	Španiel, Majesky, Kolarčik	14.07.2007	ZA-58562	42° 7' 40.512"	23° 10' 5.484"	no	no		yes
		Aurinia	*saxatilis*	Romania	Podişul Dobrogei, Cheia	Španiel, Marhold, Lihová, Kolarčik	18.05.2007	ZA-58560	44° 30' 27.792"	28° 25' 39.288"	no	no		yes
		Aurinia	*saxatilis*	Serbia	Mt Radan, Sokolov Vis	Niketić, Tomović	01.05.2008	ZA-59650	43° 59' 5.352"	21° 26' 58.2"	no	no		yes
		Aurinia	*saxatilis*	Serbia	Svrljiški Timok gorge, Svrljin Grad ruin	Niketić, Tomović	s.n.	ZA-59651	43° 28' 20.928"	22° 5' 43.656"	no	no		yes
		Aurinia	*saxatilis*	North Macedonia	Ohrid	Rešetnik	14.07.2009	ZA-58531	41° 7' 17.832"	20° 49' 9.984"	no	no		yes
		Aurinia	*saxatilis*	North Macedonia	Mavrovo NP, Ribnica	Kolář	09.06.2014	no voucher	41° 43' 13.08"	20° 36' 34.92"	no	no		yes
		Aurinia	*saxatilis*	Greece	Olympus Mt, road Leptokarya - Karya	Niketić, Tomović, Đurović, Buzurović	07.08.2014	no voucher	40° 1' 29.14"	22° 29' 16.01"	no	no		yes
		Aurinia	*saxatilis*	Greece	Kozani	Niketić, Tomović, Đurović, Buzurović	12.08.2014	ZA-59535	40° 14' 32.316"	21° 58' 12.144"	no	no		yes
		Aurinia	*saxatilis*	Romania	Bicaz Chei, Cheile Sugaului gorge	Kolář	25.08.2015	no voucher	46° 49' 30.612"	25° 51' 1.8"	no	no		yes
		Aurinia	*saxatilis*	Serbia	Niš, Jelašnička klisura	Bogdanović, Ljubičić, Rešetnik, Terlević	20.06.2019	ZA-60654	43° 16' 54.12"	22° 3' 52.092"	no	no		yes
		Aurinia	*saxatilis*	North Macedonia	Šar-Planina, Rashche	Bogdanović, Doboš, Grgurev, Rešetnik	11.07.2019	no voucher	42° 2' 14.64"	21° 15' 4.72"	no	no		yes
		Aurinia	*saxatilis*	North Macedonia	Ohridsko jezero, Gradišće	Bogdanović, Doboš, Grgurev, Rešetnik	12.07.2019	no voucher	40° 59' 15.04"	20° 48' 0.79"	no	no		yes
		Aurinia	*saxatilis*	North Macedonia	Korab Mt, Strezimir, Stirovica canyon	Bogdanović, Doboš, Grgurev, Rešetnik	13.07.2019	ZA-59320	41° 47' 58.524"	20° 37' 40.836"	no	no		yes
		Aurinia	*saxatilis*	North Macedonia	Markovi Kuli, Skopje - Novachani	Bogdanović, Grgurev, Rešetnik	16.07.2019	no voucher	41° 49' 54.77"	21° 41' 28.28"	no	no		yes
		Aurinia	*saxatilis*	North Macedonia	Skopska Crna Gora, Brodec	Bogdanović, Grgurev, Rešetnik	18.07.2019	ZA-59306	42° 9' 15.012"	21° 27' 0.072"	no	no		yes
		Aurinia	*saxatilis*	Greece	Island Kefalonia, Mousata	Bogdanović, Brullo, Cambria	26.09.2019	no voucher	38° 8' 36.17"	20° 36' 43.09"	no	no		yes
		Aurinia	*saxatilis*	Greece	Mt Hymettus, Vigor	Bogdanović, Brullo, Cambria	29.09.2019	no voucher	37° 57' 23.076"	23° 48' 57.852"	no	no		yes

Supplementary Table 2. Genbank accession numbers of previously published plastid sequences of the tribe Alysseae used in phylogenetic analyses.

Species	GenBank
Acuston lunarioides (Willd.) Raf.	KF022974
Alyssoides utriculata (L.) Medik.	KF022859
Alyssum alyssoides (L.) L.	KF022863
Alyssum aurantiacum Boiss.	KF022866
Alyssum baumgartnerianum Bornm. ex Jos.Baumgartner	KF022867
Alyssum dasycarpum Stephan ex Willd.	KF022873
Alyssum dasycarpum Stephan ex Willd.	KF022874
Alyssum diffusum subsp. *garganicum* Španiel, Marhold, N.G.Passal. & Lihová	KF022877
Alyssum doerfleri Degen	KF022878
Alyssum fastigiatum Heywood	KF022907
Alyssum granatense Boiss. & Reut.	KF022880
Alyssum harputicum T.R.Dudley	KF022881
Alyssum hirsutum M.Bieb.	KF022882
Alyssum misirdalianum Orcan & Binzet	KF022892
Alyssum persicum Boiss.	KF022912
Alyssum pluscanescens (Raim. ex Jos.Baumgartner) Španiel, Lihová & Marhold	KF022902
Alyssum strigosum Banks & Sol.	KF022925
Alyssum strigosum Banks & Sol.	KF022924
Alyssum turkestanicum Regel & Schmalh.	KF022931
Alyssum turkestanicum Regel & Schmalh.	KF022875
Aurinia corymbosa Griseb.	KF022933
Aurinia gionae (Quézel & Contandr.) Greuter & Burdet	KF022935
Aurinia leucadea (Guss.) K.Koch	KF022937
Aurinia moreana Tzanoud. & Iatroú	KF022942
Aurinia petraea (Ard.) Schur	KF022943
Aurinia sinuata (L.) Griseb.	KF022954
Berteroa incana (L.) DC.	KF022955
Berteroa mutabilis (Vent.) DC.	KF022956
Berteroa obliqua (Sm.) DC.	KF022957
Berteroa orbiculata DC.	KF022958
Bornmuellera baldaccii (Degen) Heywood	KF022959
Bornmuellera cappadocica (Willd.) Cullen & T.R.Dudley	KF022960
Bornmuellera dieckii Degen	KF022962
Bornmuellera emarginata (Boiss.) Rešetnik	KF022989
Bornmuellera tymphaea (Hausskn.) Hausskn.	KF022963
Brachypus suffruticosus (Vent.) V.I.Dorof.	KF022978
Clypeola aspera (Grauer) Turrill	KF022967
Clypeola dichotoma Boiss.	KF022968

Clypeola jonthlaspi L.	KF022969
Cuprella homalocarpa (Fisch. & C.A.Mey.) Salmerón-Sánchez, Mota & Fuertes	KF022884
Degenia velebitica (Degen) Hayek	KF022970
Fibigia clypeata (L.) Medik.	KF022972
Fibigia macrocarpa (Boiss.) Boiss.	KF022976
Galitzkya macrocarpa (Ikonn.-Gal.) V.V.Botschantz.	KF022981
Galitzkya macrocarpa (Ikonn.-Gal.) V.V.Botschantz.	KF022981
Galitzkya potaninii (Maxim) V.V.Botschantz.	KF022983
Hormathophylla cadevalliana (Pau) T.R.Dudley	KF022984
Hormathophylla lapeyrouseana (Jord.) P.Küpfer	KF022986
Hormathophylla spinosa (L.) P.Küpfer	KF022987
Irania umbellata (Boiss.) Hadač & Chrtek	KF022980
Lepidotrichum uechtritzianum (Bornm.) Velen. & Bornm.	KF022988
Lepidotrichum uechtritzianum (Bornm.) Velen. & Bornm.	KF022988
Meniocus linifolius (Stephan ex Willd.) DC.	KF022887
Odontarrhena condensata (Boiss. & Hausskn.) Jord. & Fourr.	KF022870
Odontarrhena corymbosoidea (Formánek) Španiel, Al-Shehbaz, D.A.German & Marhold	KF022872
Odontarrhena metajnae (Plazibat) Španiel, Al-Shehbaz, D.A.German & Marhold	KF022917
Odontarrhena nebrodensis (Tineo) L.Cecchi & Selvi	KF022906
Odontarrhena serpyllifolia (Desf.) Jord. & Fourr.	KF022916
Phyllolepidum cyclocarpum (Boiss.) L.Cecchi	KF022990
Phyllolepidum rupestre (Sweet) Trinajstić	KF022991
Physoptychis caspica (Hablitz) V.V.Botschantz.	KF022994
Resetnikia triquetra (DC.) Španiel, Al-Shehbaz, D.A.German & Marhold	KF022979

Supplementary Table 3. Bioclimatic variables with their definitions (following worldclim.org) used for species distribution modelling. Variables in bold are variables used in modelling after accounting for multicollinearity.

variable name	description
**bio04**	**temperature seasonality**
bio05	mean daily maximum air temperature of the warmest month
bio06	mean daily minimum air temperature of the coldest month
**bio10**	**mean daily mean air temperatures of the warmest quarter**
**bio13**	**precipitation amount of the wettest month**
bio14	precipitation amount of the driest month
**bio17**	**mean monthly precipitation amount of the driest quarter**
**bio18**	**mean monthly precipitation amount of the warmest quarter**

Supplementary Table 4. Characteristics of different RADseq datasets used in various analyses, including number of investigated individuals (N_{ind}), populations (N_{pop}) and variants ($N_{variants}$).

**Analysis**	N_{ind}	N_{pop}	$N_{variants}$
RaxML (incl. outgroup)	181	64	27791
SNAPP - reduced entire dataset (excl. outgroup)	56	56	1914
fastSTRUCTURE, NeighbourNet - entire dataset (excl. outgroup)	180	63	7005
fastSTRUCTURE - South Balkan - Apennine Group	74	26	6325
fastSTRUCTURE - Central Balkan Group	54	19	4804
fastSTRUCTURE - Central European and East Balkan-Carpathian Group	43	15	3338
PCoA	180	63	2279
Dadi – Core Central Balkan Subgroup vs. East Balkan- Carpathian Group	24/16	2	451/337
Dadi - East Balkan- Carpathian Group vs. Central European Group	16/18	2	337/235

Supplementary Table 5. Results of all 2D demographic models tested for pairwise population comparisons between (i) Core Central Balkan Subgroup and East Balkan-Carpathian Group, (ii) East Balkan-Carpathian Group and Central European Group and (iii) Central European Group and East Balkan-Carpathian Group. All models tested are visualised and provided with full and abbreviated names in Supplementary Figure 2. Other abbreviations are as follows: AIC, Akaike information criterion; ΔAIC, difference in AIC to the best-scoring model of the comparison of two particular groups; ωi, Akaike weight; theta, the effective mutation rate of the reference population (fully defined in the caption of Table 2).

**Dadi - two population Island models**							
	Model	log-likelihood	AIC	Δ AIC	ω	chi-squared	theta
**Core Central Balkan Subgroup vs. East Balkan-Carpathian Group**	**founder_sec_contact_asym_two_epoch**	**-238.33**	**492.66**	**0**	**0.817**	**152.33**	**331.45**
	vic_sec_contact_asym_mig	-240.42	496.84	4.18	0.101	148.78	171.16
	founder_asym	-241.98	497.96	5.3	0.058	154.51	1118.68
	founder_anc_asym_two_epoch	-241.87	499.74	7.08	0.024	158.42	47.26
	founder_nomig	-259.99	529.98	37.32	0.000	242.08	826.32
	vic_no_mig	-259.84	529.68	37.02	0.000	279.33	145.76
	founder_no_mig_two_epoch	-260.46	532.92	40.26	0.000	253.37	140.54
	vic_anc_asym_mig	-259.86	535.72	43.06	0.000	262.07	176.95
**East Balkan-Carpathian Group vs. Central European Group**							
	**vic_no_mig**	**-177.32**	**364.64**	**0**	**0.427**	**42.8**	**96.62**
	founder_anc_asym_two_epoch	-175.33	366.66	2.02	0.156	31.26	458.67
	founder_nomig	-178.49	366.98	2.34	0.133	41.76	15.46
	founder_sec_contact_asym_two_epoch	-175.96	367.92	3.28	0.083	35.06	296.63
	vic_anc_asym_mig	-175.87	367.74	3.1	0.091	35.87	93.78
	founder_asym	-177.37	368.74	4.1	0.055	36.78	47.84
	founder_no_mig_two_epoch	-178.34	368.68	4.04	0.057	42.68	8.95
	vic_sec_contact_asym_mig	-200.01	416.02	51.38	0.000	82.52	599.21
**Central European Group vs. East Balkan-Carpathian Group**							
	**founder_anc_asym_two_epoch**	**-187.45**	**390.9**	**0**	**0.685**	**26.66**	**61.51**
	founder_asym	-189.48	392.96	2.06	0.244	31.8	127.91
	founder_nomig	-192.94	395.88	4.98	0.057	46.15	79.01
	founder_no_mig_two_epoch	-193.33	398.66	7.76	0.014	46.9	57.39
	vic_anc_asym_mig	-201.74	419.48	28.58	0.000	56.64	29.21
	vic_no_mig	-212.02	434.04	43.14	0.000	83.84	118.88
	founder_sec_contact_asym_two_epoch	-220.18	456.36	65.46	0.000	82	355.6
	vic_sec_contact_asym_mig	-222.85	461.7	70.8	0.000	101.41	335.77

Supplementary Table 6. Overall model scores showing AUC values, sensitivity, and specificity for each species distribution model developed.

	**AUC**	**Sensitivity**	**Specificity**
Total dataset	0,957	96,703	83,779
Central Balkan, East Balkan-Carpathian and Central European Group	0,963	97,619	85,731
South Balkan-Apennine Group	0,984	100	93,584
